# Transcriptome Analysis of Deltamethrin Resistance Mechanisms in *Aedes aegypti* Populations from Metro Manila, Philippines

**DOI:** 10.3390/genes17091093

**Published:** 2026-09-10

**Authors:** Marc Ralph M. Solomon, Ma. Anita M. Bautista

**Affiliations:** Functional Genomics Laboratory, National Institute of Molecular Biology and Biotechnology, College of Science, University of the Philippines Diliman, Quezon City 1101, Philippines; mmsolomon1@alumni.up.edu.ph

**Keywords:** *Aedes aegypti*, insecticide resistance, deltamethrin, transcriptomics, RNA-Seq, detoxification, Philippines

## Abstract

**Background**: Arboviral transmission by *Aedes aegypti* is exacerbated by insecticide resistance, which undermines effective vector control strategies. In the Philippines, deltamethrin resistance in several *Ae. aegypti* populations has been reported and prompts an investigation into the possible molecular mechanisms involved. **Methods**: Two *Ae. aegypti* populations were subjected to susceptibility assays to deltamethrin and were RNA-sequenced for transcriptome analysis. **Results**: The two (2) *Ae. aegypti* populations were resistant to deltamethrin and had LC_50_ values of 1.26 and 2.15 μg/mL at the zeroth generation (G0) that shifted to 2.21 and 2.08 μg/mL at the second generation (G2) after sublethal deltamethrin exposure, although not statistically significant. Relative to a control population unexposed to any insecticide, DGE analysis of the RNA-Seq data revealed significant upregulation of cytochrome P450, glutathione S-transferase, and carboxyl/cholinesterase genes in resistant populations. Moreover, GO and KEGG analyses revealed enrichment of cytochrome P450-related terms and xenobiotic metabolism pathways. **Conclusions**: RNA-Seq analysis results of selected genes show concordance with their respective gene expression measured via qPCR (R = 0.89; *p* = 0.00049). This study provides insights into the possible molecular mechanisms of deltamethrin resistance in Philippine *Ae. aegypti* populations, providing informed decision-making in vector management.

## 1. Introduction

*Aedes* mosquitoes are the primary vectors of arboviruses such as Zika, Chikungunya, yellow fever, and dengue, which confer diseases that remain a global public health concern due to the increasing number of infections, morbidity, and mortality, owing to their vectors’ wide global distribution, high vector competence, frequent human bites, and survival in urban areas [1,2,3]. To date, there are still no reliable and efficient treatments, such as vaccines, for these arboviral diseases; hence, controlling the population of their insect vectors has been the primary strategy to prevent transmission and outbreaks [4]. The most common vector control strategy is the use of insecticides, particularly pyrethroids, which are extensively used among other insecticides owing to their low mammalian toxicity and high insecticidal effectiveness [5]. Pyrethroids are preferred active ingredients for indoor residual spraying (IRS) and long-lasting insecticidal nets, hence accounting for the majority of IRS coverage worldwide [6,7]. In the Philippines, where dengue is endemic, synthetic pyrethroids are commonly used for regular insecticidal fogging during outbreaks and after the rainy season [8,9].

Despite ongoing efforts to curb arboviral disease transmission using insecticides, more than 110,000 cases (437 deaths) were documented in the Philippines from January to May 2025, with 19,572 cases coming from Metro Manila (216% increase vs. 2024) [10]. Continuous incidences of infections are being linked by several studies to the overuse and improper application of pyrethroids, leading to increased selection pressure for the development of pyrethroid-resistant *Aedes* mosquitoes [7]. The World Health Organization pointed out that heavy reliance on pyrethroids has significantly increased resistance in *Ae. aegypti*, putting global efforts at risk of becoming ineffective [6]. Given that this dilemma promotes the transmission of mosquito-borne diseases, comprehensive surveillance and monitoring of insecticide resistance in the Philippines is imperative to reformulate the use and application of pyrethroids. However, studies tackling insecticide resistance in the Philippines are scarce [11,12].

There are two (2) known pyrethroid resistance mechanisms previously identified in *Ae. aegypti*, namely: target-site insensitivity from structural modifications in the insecticide’s target proteins, and increased expression of metabolic detoxification enzymes [13]. Non-synonymous mutations in the voltage-gated sodium channel (*vgsc*) gene result in target-site resistance, which confers reduced neuronal sensitivity of mosquitoes to insecticides [14]. Meanwhile, induction of detoxification genes enhances the expression levels of cytochrome P450 monooxygenases (P450s or *CYP* for genes), carboxy/cholinesterases, glutathione S-transferases (GSTs), and UDP-glycosyltransferases, which in turn promote insecticide metabolism [15]. Mutations resulting in this type of resistance, however, remain elusive because there are approximately 160 *CYP*s in *Aedes* mosquitoes, and the factors involved in *CYP* gene expression regulation are largely unidentified [2].

Among the pyrethroids, deltamethrin is widely used in Southeast Asian countries, including Malaysia, Singapore, Indonesia, and Thailand [4]. Due to its long history of use in the region, several studies have reported the development of resistance in *Ae. aegypti* to deltamethrin [16,17,18,19,20]. Underlying resistance mechanisms reported in these studies include target-site mutations, metabolic detoxification, reduced insecticide penetration through the mosquito cuticle, and behavioral changes [5,21]. In the Philippines, the information on insecticide resistance in *Ae. aegypti* is based on a susceptibility study in Manila [11], a pyrethroid susceptibility study in *Ae. aegypti* in Cebu City [12], and a report of insecticide resistance in *Ae. aegypti* in Metro Manila [22]. However, the molecular basis of resistance in these reports remains understudied; hence, this study sought to identify and characterize the underlying molecular mechanism of pyrethroid resistance in the Philippine populations of *Ae. aegypti* by sequencing and analyzing the transcriptomes of deltamethrin-resistant populations.

## 2. Materials and Methods

### 2.1. Mosquito Collection

Mosquito eggs were sourced from the collections of Philippines’ Department of Health—Metro Manila Center for Health Development (DOH-MMCHD), obtained using ovitraps placed indoors and outdoors of selected households in Barangay Manggahan, Pasig City (PG13) (latitude from 14.6051° N to 14.59335° N and longitude 121.09304° E to 121.10635° N; 9–12 m above sea level), and Barangay Moonwalk, Parañaque City (PE07) (latitude from 14.49217° N to 14.48983° N and longitude from 121.01825° E to 121.02006° E; 12–13 m above sea level), both located in Metro Manila, during the month of June 2024. These sites were selected based on dengue incidence and deltamethrin usage in Metro Manila. Wooden paddles containing eggs were collected and transported to the insectary (ACL-4/BSL-2) of the National Institute of Molecular Biology and Biotechnology, University of the Philippines (UP) Diliman, for rearing and maintenance following the guidelines from the Joint Food and Agriculture Organization and International Atomic Energy Agency (FAO/IAEA) [23]. Larvae were fed with Brewer’s yeast, and rearing conditions were maintained at 27 ± 1 °C, 70–80% relative humidity, and a photoperiod of 12 h of light and 10 h of dark cycles, with one (1) hour each for dusk and dawn, inside a Fitotron^®^ SGC2 insect growth chamber (Weiss Technik GmbH, Reiskirchen Germany). Emerged adult mosquitoes in rearing cages were maintained at 26 ± 2 °C. Adult *Ae. aegypti* mosquitoes were maintained with a supply of cotton soaked in 10% honey solution.

### 2.2. Deltamethrin Susceptibility Bioassay and Rearing of Deltamethrin-Resistant Mosquitoes

Susceptibility to deltamethrin of *Ae. aegypti* field-collected mosquitoes from PG13 and PE07 was determined by performing the CONUS bottle bioassay protocol of the US Centers for Disease Control and Prevention (CDC) [24]. From this point, adult mosquitoes that emerged directly from eggs collected from the field (PG13 and PE07), or the zeroth generation (G0), are called Field-G0. For each population, six (6) Wheaton bottles were used, four (4) of which were insecticide-treated and two (2) were controls. Insecticide-treated bottles were coated internally with deltamethrin at a diagnostic concentration (0.75 μg/mL) prepared using absolute ethanol AR (Analytical Reagent Grade) (RCI Labscan Limited, Bangkok, Thailand), whereas the control bottles were coated only with absolute ethanol AR. Using a pooter, 20–25 non-blood-fed mosquitoes were introduced into each bottle. The number of alive and dead mosquitoes was counted upon release (time 0) and at the 30 min mark diagnostic time. Based on calculated percent mortalities, susceptibility of each population was determined following the phenotypic resistance frequency assessment guide from WHO presented in Table 1 [25].

To validate the susceptibility results from the CDC CONUS bioassay, *Ae. aegypti* eggs were collected from PG13 and PE07 in November 2024 and were reared to adulthood for WHO’s insecticide susceptibility tube testing, which was performed using 0.03% deltamethrin in silicone oil impregnated on filter papers [25]. The test was performed by introducing 20–25 non-blood-fed female mosquitoes into each tube with impregnated paper (5 replicates) and allowing exposure for one (1) hour. The number of dead and alive mosquitoes was counted after a 24 h recovery period. Two control tubes were used containing filter paper impregnated with silicone oil only.

Resistance level was determined through median lethal concentration (LC_50_)-based modified bottle bioassay [26]. Seven concentrations with two (2) replicates each, including control, were prepared by 10-fold serial dilution of deltamethrin in absolute ethanol AR, producing the following concentrations: 0, 0.0075, 0.075, 0.75, 7.5, 75, and 750 μg/mL. The mosquitoes were exposed for one (1) hour and transferred afterward to untreated bottles for a 24 h recovery. Computed percent mortalities for each concentration after the recovery period were plotted for Probit analysis using the BioRssay package [27] in RStudio (version 2024.12.0+467) to obtain LC_50_ values.

Resistant and deltamethrin-exposed Field-G0 adults that survived the assays were then allowed to lay eggs to perform further rearing with sublethal exposure to deltamethrin for two (2) generations (G2), generating Field-G2 adult mosquitoes. Exposures were performed to every generation of each population using their respective LC_25_ values calculated from the baseline (G0) LC_50_ assay results of PG13 and PE07. Surviving female and male mosquitoes (3:1) were allowed to mate for 24 h. Female mosquitoes were then fed with defibrinated pig’s blood from a slaughterhouse twice a week using an artificial feeder made of corrugated aluminum sheets covered with parafilm. Two days after blood feeding, black plastic cups lined with filter paper and filled 1/3 with seasoned (left to stand for 24 h) tap water were placed inside the cages and left for three (3) days to allow blood-fed female mosquitoes to oviposit. Filter papers with eggs were air-dried and stored in containers for subsequent rearing.

### 2.3. RNA Sequencing

Total RNA was extracted from PG13 and PE07 *Ae. aegypti* mosquitoes, i.e., both from G0 and G2 subpopulations (reared from June 2024 collection) using TRIzol reagent (Invitrogen, Carlsbad, CA, USA). In order to perform differential gene expression analysis, RNA samples were also extracted from a population from the University of the Philippines Los Baños, Laguna, Philippines (UPLB-UNXP-G122) that has been reared in the laboratory for 122 generations and served as a control or reference population, based on the assumption that its gene expression profiles are at the basal level. Each RNA sample was a pool of five (5) non-blood-fed female mosquitoes [28]. Potential genomic DNA contamination was removed by TURBO DNase I (Invitrogen, Carlsbad, CA, USA). DNase-treated RNA extracts were checked for integrity, purity (A260/A280), and quantity using agarose gel electrophoresis (AGE), UV-Vis spectrophotometry using Jenway^®^ Spectrophotometer 7415 (Cole-Parmer, Vernon Hills, IL, USA), and fluorometry using Qubit^TM^ RNA BR protocol in Qubit^TM^ fluorometer (Invitrogen, Thermo Fisher Scientific, Waltham, MA, USA), respectively.

Prior to library preparation, we checked the RNA integrity number (RIN) of each sample using an Agilent 2200 TapeStation System (Agilent, Palo Alto, CA, USA). Sample concentrations were normalized to at least 1000 ng using nuclease-free water (NFW), creating final sample volumes of 50 μL. Using TruSeq RNA Library Prep Kit v2 (Illumina, San Diego, CA, USA), the cDNA libraries of the RNA samples were prepared following the manufacturer’s protocol. Libraries were quantified and checked for quality and then normalized to a 4 nM concentration using 10 mM Tris-HCl (pH 8.5) with 0.1% Tween 20. Libraries were then pooled, diluted to a final loading concentration of 400 pM, and loaded onto the NovaSeq^TM^ 6000 Sequencing System (Illumina, CA, USA) with a NovaSeq^TM^ SP flow cell, creating 100 bp paired-end reads. Sequencing was done at the Philippine Genome Center, University of the Philippines Diliman, Quezon City, Metro Manila, Philippines. The Illumina sequencing datasets were deposited in NCBI’s Gene Expression Omnibus (GEO) under Series accession number GSE305050, with raw reads archived in the Sequence Read Archive (SRA) under BioProject ID PRJNA1304288.

### 2.4. Transcriptome Analysis

FASTQ sequencing reads were demultiplexed, trimmed to remove adapter sequences, and filtered based on a Phred quality score of 30 (Q30) through the sequencer-on-board NovaSeq^TM^ Control Software v.1.8 (Illumina, CA, USA). Read qualities in each sample were assessed using FastQC v.0.12.1 [29], and those with scores less than 33 and lengths lower than 35 bp were trimmed using Trimmomatic v.0.40 [30]. Trimmed sequences were sorted and indexed by Samtools v.1.20 [31] and were mapped and aligned to the annotated reference genome of *Ae. aegypti* (GCA_002204515.1) AaegL5.3 [32] available in Vector Base database (https://vectorbase.org/vectorbase/app/, accessed on 7 April 2026) using STAR v.2.7.11b [33]. Features of the mapped reads to detect biases in sequencing and/or mapping were assessed using QualiMap v.2.3 [34]. Consensus transcriptome of all the samples was also assembled using Cufflinks v.2.2.1 [35] and StringTie v.3.0.0 [36]. Completeness of the assembly outputs from Cufflinks and StringTie was compared using Benchmarking Universal Single-Copy Orthologs (BUSCO) v.5.8.2 [37] in transcriptome mode using the diptera_odb10 (20 November 2019) lineage dataset with 3285 BUSCO markers. Prior to BUSCO assessment, output assemblies were filtered first using CD-HIT v.4.8.1 [38] to cluster similar sequences into representative transcripts to reduce redundancy and avoid artificial inflation in BUSCO completeness scores.

To identify differentially expressed genes (DEGs), gene counts were generated using featureCounts v.2.0.8 [39]. Counts of each gene were size-normalized and analyzed for differential gene expression using DESeq2 v.1.46.0 [40] in RStudio (version 2024.12.0+467), with counts from the UPLB-UNXP-G122 population used as the reference for the relative expression of genes in the Field-G0 and Field-G2 populations. Using the lfcShrink() wrapper function of DESeq2, apeglm (Approximate Posterior Estimation for GLM) was called to perform Bayesian shrinkage estimation of logarithmic fold changes (LFCs) to minimize false positives from noisy low-count genes [41,42]. The *p*-values were also adjusted using the Benjamini–Hochberg (BH) method to account for false discovery rates (FDR = 0.1) (32,33).

Gene ontology (GO) and Kyoto Encyclopedia of Genes and Genomes (KEGG) pathway enrichment analyses were performed on the DEGs with LFC greater than 1.0 and FDR-corrected *p*-values less than 0.10 using the g:Profiler2 package v.0.2.3 [43] in RStudio (version 2024.12.0+467). Using the *Ae. aegypti* genome assembly and annotation (AaegL5.3), upregulated DEGs were analyzed by categorizing them based on GO terms for biological processes (BP), molecular functions (MF), and cellular components (CC), as well as their association with KEGG pathways.

### 2.5. Quantitative PCR Validation

Expressions of selected DEGs from the RNA-Seq results of the Field-G2 population were checked using quantitative PCR (qPCR) to confirm the validity of the DGE analysis. Primers were designed using NCBI’s Primer-BLAST (https://www.ncbi.nlm.nih.gov/tools/primer-blast/, accessed on 7 April 2026). Ribosomal protein L32 (*rp49*), with primer sequences: forward 5′-ACAAGCTTGCCCCCAACT-3′ and reverse 5′-CCGTAACCGATGTTTGGC-3′, was used as an internal control with an annealing temperature of 60 °C and a product size of 97 bp [44]. First-strand cDNA from the Field-G2 RNA samples was prepared using SuperScript™ III First-Strand Synthesis System (Invitrogen, MA, USA), following the manufacturer’s protocol. Using iTaq^TM^ Universal SYBR^®^ Green Supermix (Bio-Rad Laboratories, Inc., Hercules, CA, USA), qPCR reaction mix for each sample was prepared by creating 10-μL reactions composed of 2.5 μL NFW, 0.25 μL each of forward and reverse primers (10 μM), 5 μL iTaq^TM^ Universal SYBR^®^ Green Supermix (2X), and 2 μL cDNA Template (20 ng/μL). qPCR reactions were incubated in CFX Opus Real-time PCR System (Bio-Rad Laboratories, Inc., CA, USA), programmed with the following qPCR conditions using CFX Maestro Software (Bio-Rad Laboratories, Inc., CA, USA, version 2.3): initial denaturation at 95 °C for 5 min, 40 cycles of denaturation at 95 °C for 10 s and annealing and extension at 60 °C for 40 s, followed by final extension at 72 °C for 10 s, and finally melt curve analysis from 65 to 95 °C for 5 min.

Standard curves were generated by amplifying each DEG in four (4) standard concentrations (0.02, 0.20, 2.00, and 20.0 ng/μL) of the samples in three (3) technical replicates. These standards were prepared by pooling the cDNA of each sample that was normalized to 20 ng/μL, and then performing a 4-point 10-fold serial dilution. Using CFX Maestro Software (Bio-Rad Laboratories, Inc., CA, USA), quantification cycle (Cq) values were analyzed to generate the standard curves with computed primer efficiency (E), goodness of fit (R^2^), and slope of the best-fit line. Measurement of the expression of the selected genes was performed using cDNA samples from the Field-G2 populations, PG13 and PE07, with three (3) biological replicates for each population and three (3) technical replicates for each biological replicate. Using the primer efficiencies, mean gene expression ratios of each DEG were then manually computed using Pfaffl’s method [45], log2-transformed and compared to the fold change values from the RNA-Seq analysis. Pearson correlation coefficient was also computed from the comparison of the two (2) methods to check for concordance in the expression of the selected genes between the methods.

## 3. Results and Discussion

### 3.1. Deltamethrin-Resistance in Ae. aegypti Populations from Metro Manila

Based on the number of dead and alive mosquitoes after exposure, the following percent mortalities were computed in each assay (Table 2 and Table 3):

PG13 and PE07 *Ae. aegypti* mosquito populations (Field-G0) exhibited deltamethrin resistance, indicated by low mortality rates of 1.92% and 3.45%, respectively, in the CDC CONUS bottle bioassay and confirmed by WHO tube assay with 2.94% and 6.25% mortalities recorded, respectively. Their corresponding LC_50_ (G0) values were 1.26 and 2.15 μg/mL, respectively (Table 4). These levels of resistance could be attributed to the prolonged and repeated use of deltamethrin. Since 1997, local government units (LGUs) in Metro Manila have consistently employed pyrethroids, including deltamethrin and permethrin, for space spraying as part of their vector control strategies [22]. This long history of pyrethroid use may have subjected these *Ae. aegypti* populations to selection pressure, favoring the survival of mosquitoes that developed a resistance phenotype to pyrethroids.

The resistant PG13 and PE07 populations were reared up to the 2nd generation (Field-G2) with exposure to deltamethrin at LC_25_. The subsequent LC_50_ assay (Table 4) showed an increase in the LC_50_ of the PG13 population from 1.26 (G0) to 2.21 μg/mL (G2), suggesting a possible enhancement of resistance. However, this increase from G0 to G2 is not statistically significant due to the overlap in their corresponding confidence intervals (CIs). Similarly, the PE07 population exhibited a slight decrease in LC_50_ from 2.15 (G0) to 2.08 μg/mL (G2), which is also not significant. Although not statistically significant, it is important to acknowledge the possible biological implications of the observed trends [46]. Changes in LC_50_ following sublethal exposure may suggest a potential trend towards enhanced insecticide resistance in the field-collected *Ae. aegypti* populations. The increase could be due to molecular mechanisms activated upon exposure to insecticides like deltamethrin [47]. The wide range of CIs suggests low precision, which may be caused by the large gap between the deltamethrin concentrations used in the LC_50_ assay (10-fold increments) and biological variability among field-collected mosquitoes, possibly resulting in heterogeneous responses to deltamethrin exposure [46,48]. This limitation of the study may be addressed by using concentrations with smaller fold increments around the expected mortality range.

### 3.2. Ae. aegypti Transcriptome Assemblies with Duplicated Orthologs

Total RNA extracts obtained from the Field-G0 and Field-G2 populations of PG13 and PE07 mosquitoes, including the control population UPLB-UNXP-G122 (Table S1), exhibited high quality (RIN 7.2 to 9.8) (Figure S1) and were used to produce cDNA libraries. Sequencing generated 136.89 Gb of paired-end sequencing data with approximately 1.28 billion reads passing the sequencer’s internal quality filtering (Table S2) and over 97% of the base calls having quality scores (Q-scores) greater than 30 (>Q30). Qualities were consistent at >Q35 before and after trimming, which retained approximately 99% of each sample’s paired-end reads. Alignment to the *Ae. aegypti* reference genome (GCA_002204515.1) [32] showed that over 99% of the reads successfully mapped, with very low mismatch rates ranging from 0.44% to 0.47%, confirming the overall accuracy and quality of the sequencing data (Table S3) [49]. The majority of the reads (73.9% to 93.1%) mapped to single, unambiguous locations in the reference genome, exceeding the commonly accepted threshold for successful RNA-Seq alignment (>70%) [50].

**Figure 1:**
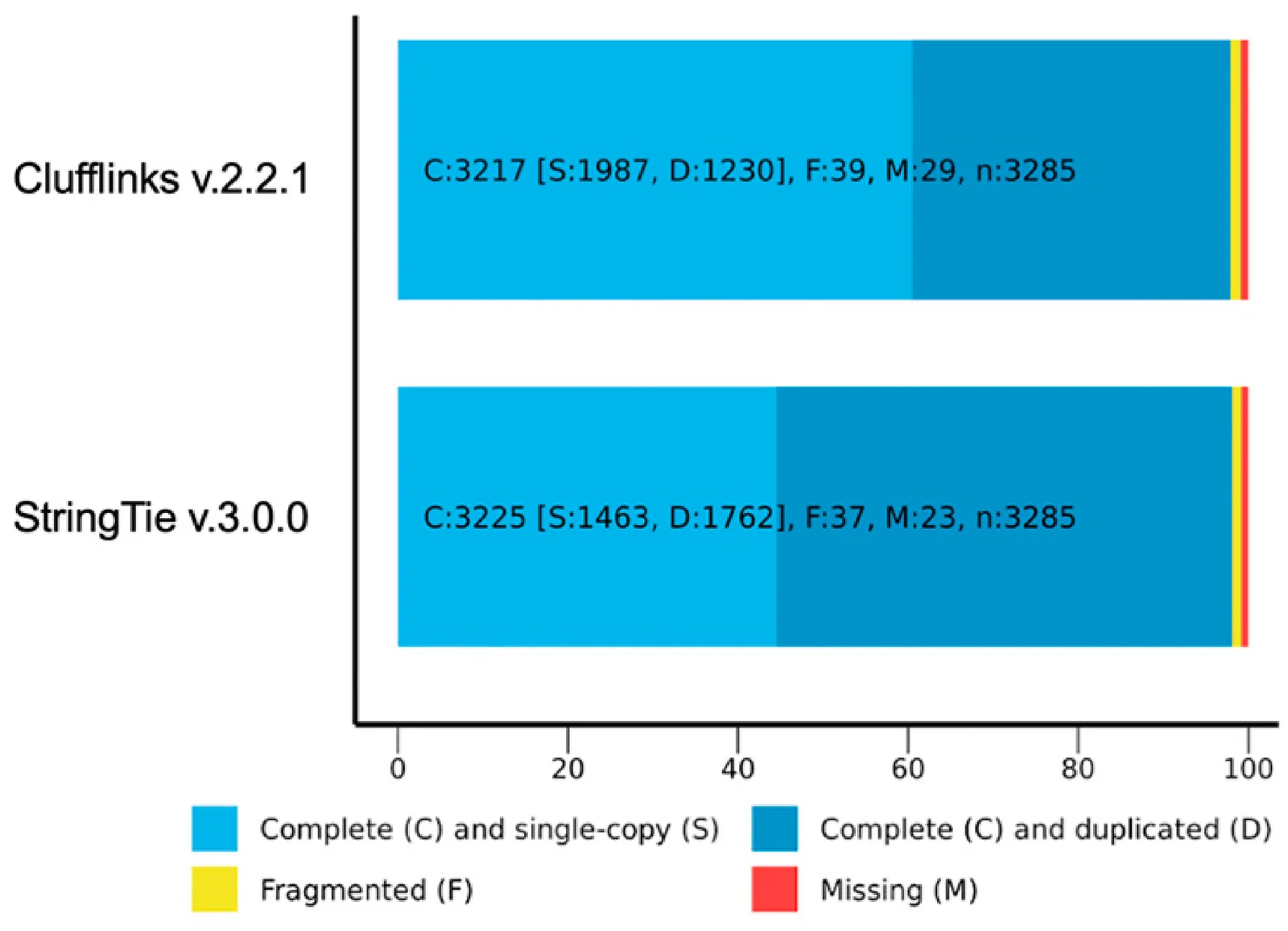
Assessment results of the transcriptome assemblies of *Aedes aegypti* generated from Cufflinks (version 2.2.1) and StringTie (version 2.17) based on Benchmarking Universal Single-Copy Orthologs (BUSCO). The top bar is from the Cufflinks assembly, and the bottom bar is from the StringTie assembly; both were filtered using CD-HIT (version 4.8.1). Results are based on 3285 BUSCO markers of the diptera_odb10 (20 November 2019) lineage dataset.

Using the annotated *Ae. aegypti* reference genome (AaegL5.3), the consensus transcriptome assemblies from all samples, as shown in Figure 1, demonstrated high completeness (~98%), with low percentages of fragmented (1.1–1.2%) and missing (0.7–0.9%) BUSCOs. Between the two (2) assemblers used, StringTie produced a slightly more complete assembly (98.2%) compared to Cufflinks (97.9%). However, Cufflinks yielded a higher proportion of complete single-copy BUSCOs (60.5%) and fewer duplicated BUSCOs (37.4%), in contrast to StringTie (44.5% single-copy; 53.6% duplicated BUSCOs). While a high proportion of duplicated BUSCOs is expected in transcriptome assemblies [51], it is notable that a substantial number of duplicates remained even after redundancy filtering. Given that the samples primarily consist of deltamethrin-resistant *Ae. aegypti* mosquitoes, the elevated level of duplicated orthologs observed may potentially reflect true biological duplications. It has been found that gene duplication events could be contributing to the overexpression of detoxification enzymes, as suggested by Bacot et al. [52], who reported that genomic duplications can play a role in enhancing the expression of genes involved in insecticide resistance in mosquitoes. Nevertheless, it is imperative to further investigate whether the observed duplicated orthologs are indeed genomic duplications contributing to overexpression of detoxification-related genes.

### 3.3. Overexpression of Detoxifying Enzymes Based on Differential Gene Expression Analysis

**Figure 2:**
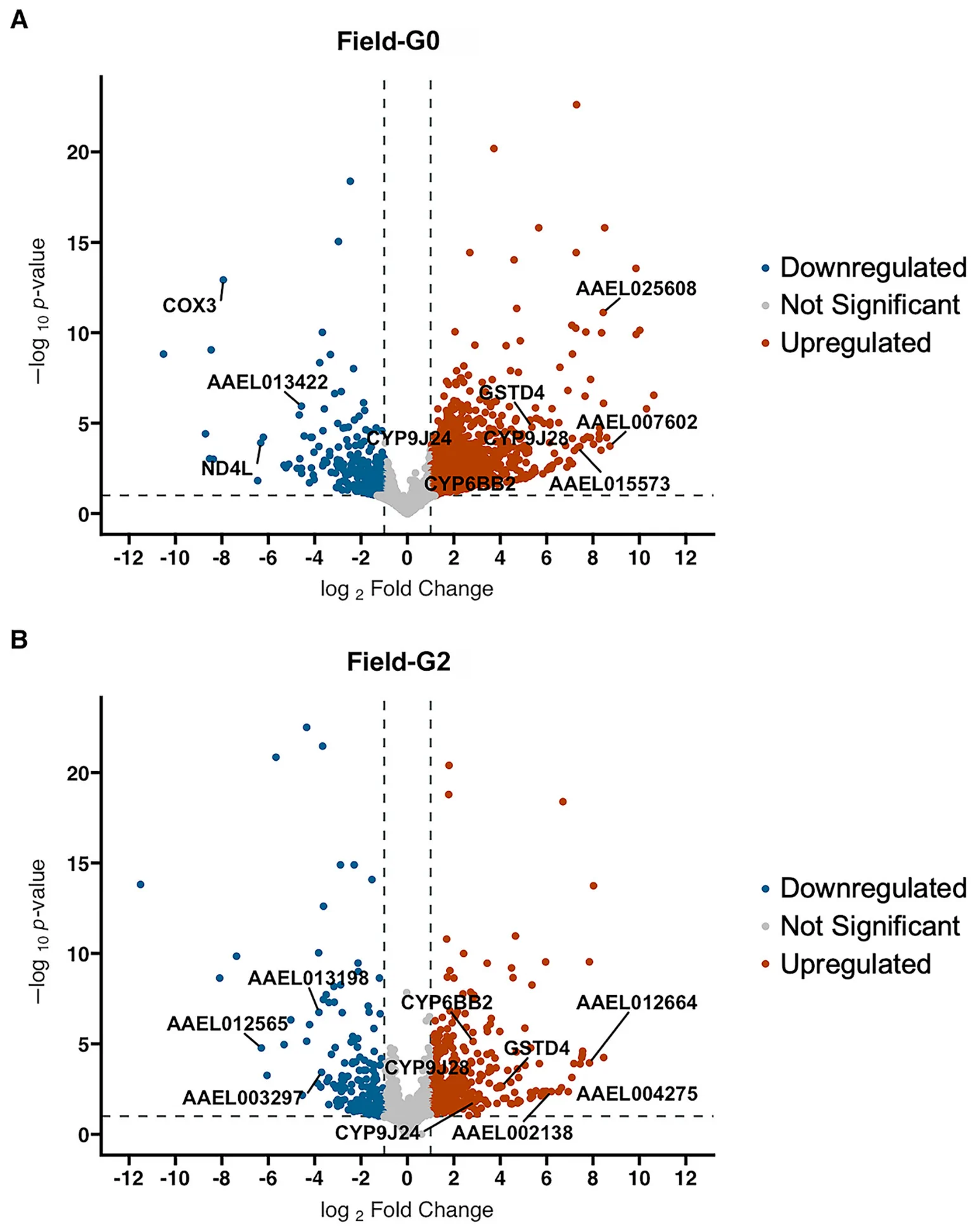
Volcano plots of differentially expressed genes (DEGs) in *Ae. aegypti* samples. (**A**) DEGs in field-collected G0 *Ae. aegypti* samples in reference to UPLB-UNXP-G122 sample; (**B**) DEGs in field-collected G2 *Ae. aegypti* samples in reference to the UPLB-UNXP-G122 sample. Red dots indicate significantly (−log_10_[*p*-value adjusted < 0.1]) upregulated (log_2_FoldChange > 1) genes; blue dots indicate significantly downregulated genes; and gray dots indicate genes with no significant difference between field and UPLB samples. The top 5 downregulated and top 5 upregulated annotated genes based on LFC are labeled. Broken-lines indicate log_2_FoldChange value and −log_10_(*p*-value adjusted) value cut-offs in x- and y-axes, respectively.

Gene-level counts revealed that at least 50% of the reads per sample were successfully assigned to annotated gene features (Figure S2), except for two (2) samples (37.3% and 29.8%, respectively) due to observed bias in their read coverage towards the 3′ end of their transcripts (NCBI GSE305050) (Figure S3). Differential gene expression (DGE) analysis comparing the Field-G0 and Field-G2 populations to the control population, UPLB-UNXP-G122, identified 3563 DEGs in the Field-G0 and 1671 DEGs in the Field-G2 population. Based on an LFC cutoff of ±1, the Field-G0 population exhibited 406 significantly downregulated DEGs (118 annotated) and 2007 significantly upregulated DEGs (764 annotated) (Figure 2A). Meanwhile, in the Field-G2 population, 174 significantly downregulated DEGs (43 annotated) and 468 significantly upregulated DEGs (164 annotated) were identified (Figure 2B). In both populations, upregulated DEGs include several genes potentially linked to metabolic insecticide resistance, notably those belonging to cytochrome P450 (*CYP*), glutathione S-transferase (*GST*), and carboxy/cholinesterase (*CCE*) gene families (Tables S4 and S5).

Most of the *CYP* genes identified belong to the *CYP9*, *CYP6*, and *CYP325* subfamilies. These *CYP*s have been previously confirmed to be present in *Ae. aegypti* through multiple studies utilizing microarray, transgenic expression, and biochemical assays [53,54,55,56]. Notably, *CYP9J28*, *CYP9J26*, *CYP9J24*, *CYP9M6*, *CYP6BB2*, and *CYP6N12* genes are consistently overexpressed in both Field-G0 and Field-G2 populations, each exhibiting LFC values greater than 2.2, corresponding to at least a 4.6-fold increase in expression. CYPs have been reported as the primary group of enzymes implicated in pyrethroid resistance in several mosquito species [57]. Generally, CYPs catalyze the oxidation of xenobiotics, producing oxidized metabolites that subsequently undergo conjugation reactions catalyzed by uridine diphosphate (UDP) glucosyltransferases (UGTs) or glucuronosyltransferases (UDPGTs) [58,59]. These conjugated products are then eliminated from mosquito cells via ATP-binding cassette (ABC) transporters [58]. Interestingly, this detoxification pathway may be supported by the current study, as UDPGT (AAEL008749) and ABC transporters (AAEL012700 and AAEL001938) were also observed to be upregulated (LFC > 1, adjusted *p* < 0.1) in both Field-G0 and Field-G2 populations (Tables S4–S7).

Most of the overexpressed *CYP* genes in this study belong to the *CYP6* and *CYP9* families. These two (2) families are part of the *CYP3* clan, which is widely recognized in insects for containing multiple-copy paralogs that are often arranged in tandem clusters on chromosomes [60]. These paralogs typically arise from gene duplication events, a mechanism increasingly implicated in metabolic resistance in mosquitoes. Recent studies have associated *CYP* gene duplications with insecticide resistance in *Ae. aegypti* [52]. Because *CYP6* and *CYP9* families are insect-specific, they may be particularly prone to evolutionary expansion through tandem duplications, leading to large genomic clusters [60,61]. Within these clusters, positive selection may act on a single gene that confers a strong detoxification advantage, like enhanced pyrethroid metabolism, and may trigger a selective sweep. This event allows neighboring *CYP* paralogs to hitchhike, increasing in frequency regardless of their individual functional contribution. If these clustered genes share *cis*-regulatory elements, co-regulation can occur, resulting in the simultaneous transcriptional activation of multiple *CYP*s. A similar phenomenon was observed in *Anopheles funestus*, where a selective sweep in a genomic region containing *CYP*s contributed to metabolic resistance against pyrethroids [62]. As the majority of upregulated *CYP*s belong to the *CYP6* and *CYP9* families, it is hypothesized that this pattern may suggest the presence of a duplication-rich genomic region under strong selective pressure from insecticide exposure. Gene duplication can increase gene dosage, resulting in elevated expression of detoxifying enzymes. This, in turn, enhances the mosquito’s capacity to metabolize and withstand insecticide exposure [63]. Taken together, this hypothesis about possible genomic duplications and selective sweeps concerning *CYP6* and *CYP9* gene families needs to be further investigated.

Similar to *CYP*s, *GST*s are also overexpressed in both populations, with LFCs ranging from 1.3 to 5.8 (2.5- to 55-fold). While most *GST*s previously associated with insecticide resistance belong to the Epsilon class [64,65], the majority of overexpressed *GST*s in this study belong to the Delta class, including *GSTD4*, *GSTD3*, and *GSTD11*. Notably, *GSTD4* has been identified as overexpressed in microarray and mRNA-Seq studies of insecticide-resistant *Ae. aegypti* [66,67]. Additionally, several *CCE*s (e.g., CCEJHE4F, AAEL001517, and CCEAE1B) are also observed to be upregulated in the Field-G0 population. The observed upregulation of *GST*s and *CCE*s, alongside *CYP*s, may reflect potential metabolic adaptations to the routine use of organophosphate- and carbamate-based insecticides in PG13 and PE07 [10]. The absence of upregulated *CCE*s in the Field-G2 population may also reflect the selective pressure exerted by two (2) generations of sublethal deltamethrin exposure, thereby favoring the expression of pyrethroid-specific resistance genes.

Between the Field-G0 and Field-G2 populations, more upregulated detoxification enzymes were identified in the Field-G0 population, while no upregulated *CCE*s were observed in the Field-G2 population. This may suggest that pyrethroid resistance in PG13 and PE07 *Ae. aegypti* may be largely driven by constitutively high expression of detoxification enzymes, particularly CYPs, rather than by induction following insecticide exposure. As such, the performed sublethal exposure indeed has not significantly elevated their expression levels any further [60]. This interpretation is corroborated by the high LC_50_ values observed in PG13 and PE07 populations at G0, which were reared without prior insecticide exposure before being subjected to the LC_50_ bioassay (Table 4). The decrease in fold change and number of upregulated *CYP*s in the Field-G2 population may also be attributed to the manner of collecting the mosquito samples after exposure. The observed decrease in LFCs and fewer upregulated *CYP*s in the Field-G2 population could also be influenced by the timing and conditions of sample collection. Specifically, mosquitoes were collected 24 h after deltamethrin exposure during the LC_50_ assay, which may have coincided with a post-exposure recovery phase. A similar trend was reported in a time-series transcriptomic analysis by Mack and Attardo [68], who observed a global downregulation of detoxification genes 24 h post-permethrin exposure in *Ae. aegypti*. The authors hypothesized that this reflected a physiological return to basal gene expression levels, potentially as part of the mosquitoes’ recovery response after insecticide-induced stress.

In addition, given the initial LC_50_ of PG13 and PE07 populations were already high (Table 4), subsequent sublethal exposures that further elevated their LC_50_ may have imposed fitness and energetic trade-offs. These trade-offs could have negatively impacted the expression of some genes, as cellular energy may have been diverted toward the expression of genes that are more efficient in conferring pyrethroid resistance or tolerance [47,69]. This hypothesis may be supported by the RNA-Seq analysis of the Field-G2 population, which showed downregulation of genes associated with energy metabolism, development, and reproduction, including glycerol kinase (AAEL008474), glucose dehydrogenase (AAEL004028), lozenge (AAEL007040), oviductin (AAEL007519), and sugar transporter (AAEL001257), among others. Lastly, the differential expression patterns observed may also be shaped by regulatory mechanisms that control the overexpression of detoxification enzymes. These mechanisms may have led to the selective activation of specific genes over others, contributing further to the mosquito populations’ adaptive responses to insecticide pressure [47].

Aside from detoxification enzymes, overexpression was also observed among genes associated with other resistance mechanisms. Notably, chitin synthase (AAEL002718) and several cuticle protein genes, which may be involved in cuticle thickening, a mechanism that reduces insecticide penetration, were upregulated in both populations [70]. Additionally, several odorant-binding proteins (OBPs) were also found to be upregulated. These OBPs are known to play roles in chemosensation, influencing excito-repellent behavior in mosquitoes, particularly by aiding in the detection and avoidance of xenobiotics such as residual insecticides [71,72]. Taking all these results together so far, with the UPLB-UNXP-G122 population used as the reference population for the DGE analysis, there is an important caveat that the observed overexpression of these genes may not be entirely because of deltamethrin resistance, but also influenced by population genetic background and long-term laboratory adaptation effects. Theoretically, this can be addressed using a susceptible *Ae. aegypti* population with a close genetic background to the field-collected populations, which was not available in the Philippines or in Metro Manila at the time of this writing.

### 3.4. Enrichment to GO Terms and KEGG Pathways Related to Metabolic Detoxification

**Figure 3:**
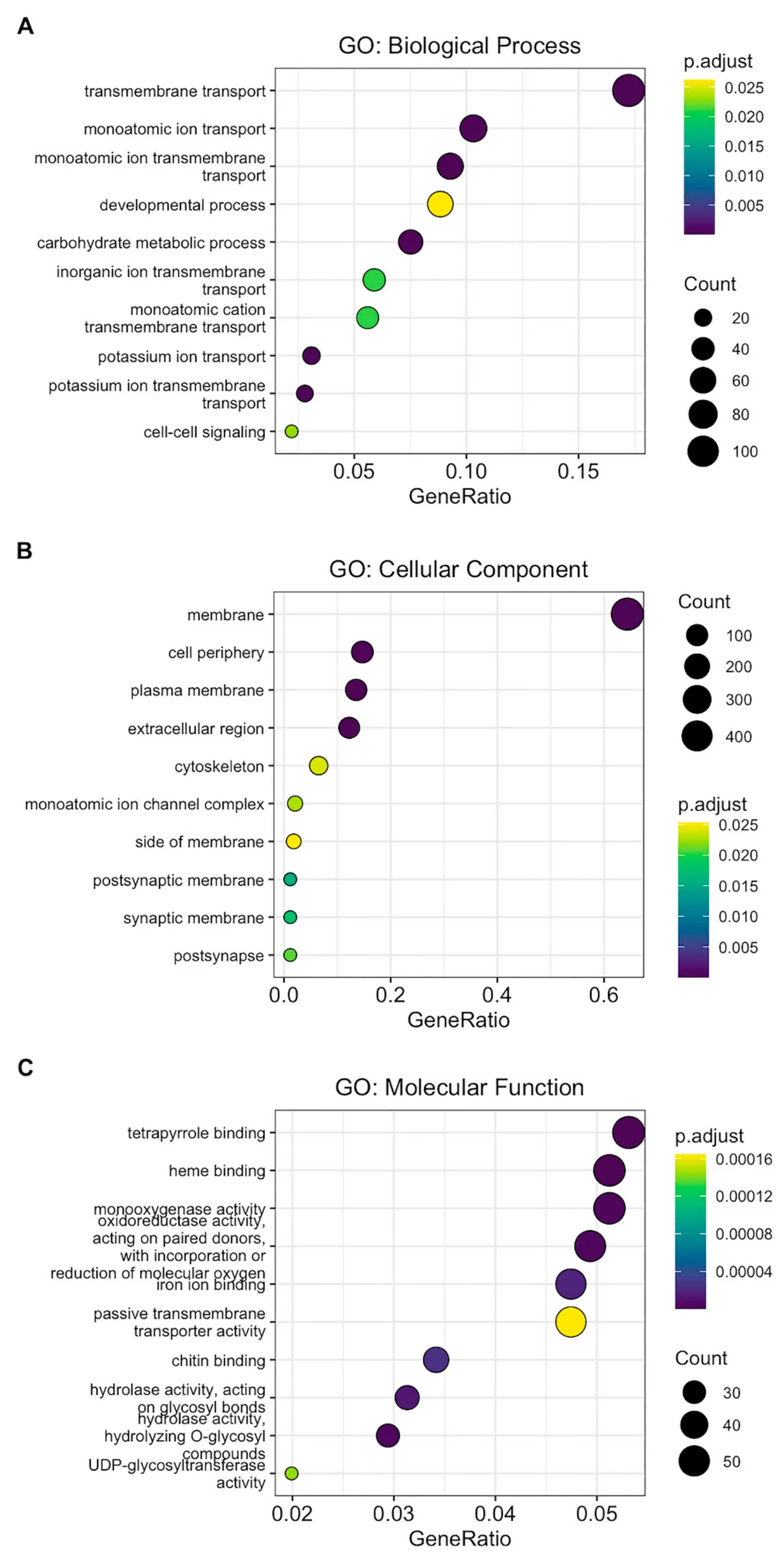
Gene function enrichment analysis of upregulated DEGs in *Ae. aegypti* Field-G0 population highlighting the top 10 enriched Gene Ontology (GO) Biological Processes (BP) terms (**A**), GO Cellular Component (CC) terms (**B**) and GO Molecular Function (MF) terms (**C**).

Further investigation of mechanisms underlying deltamethrin resistance by functional enrichment analysis of upregulated DEGs from both Field-G0 and Field-G2 populations showed significant enrichment of GO terms and KEGG pathways. In the Field-G0 population, GO:BP terms “transmembrane transport” and “monatomic ion transport” are enriched, suggesting alterations in ion channel activity or membrane permeability (Figure 3A). These may support the involvement of target-site insensitivity as a major resistance mechanism, where mutations in VGSCs result in conformational changes that impair insecticide binding and prevent prolonged depolarization [13,73]. Complementing this, enriched GO:CC terms including “membrane,” “plasma membrane,” and “postsynaptic membrane” (Figure 3B) may indicate that the upregulated DEGs are localized at or near the action site of deltamethrin, which targets membrane-bound VGSCs [74]. The enrichment of GO:MF terms such as “oxidoreductase activity,” “tetrapyrrole binding,” and other CYP-related functions may further indicate that metabolic detoxification is a primary resistance mechanism in *Ae. aegypti* (Figure 3C). Specifically, “tetrapyrrole binding”, “heme binding”, and “iron ion binding” are fundamental molecular features of CYP enzymes that enable their catalytic oxidation–reduction activity [75].

**Figure 4:**
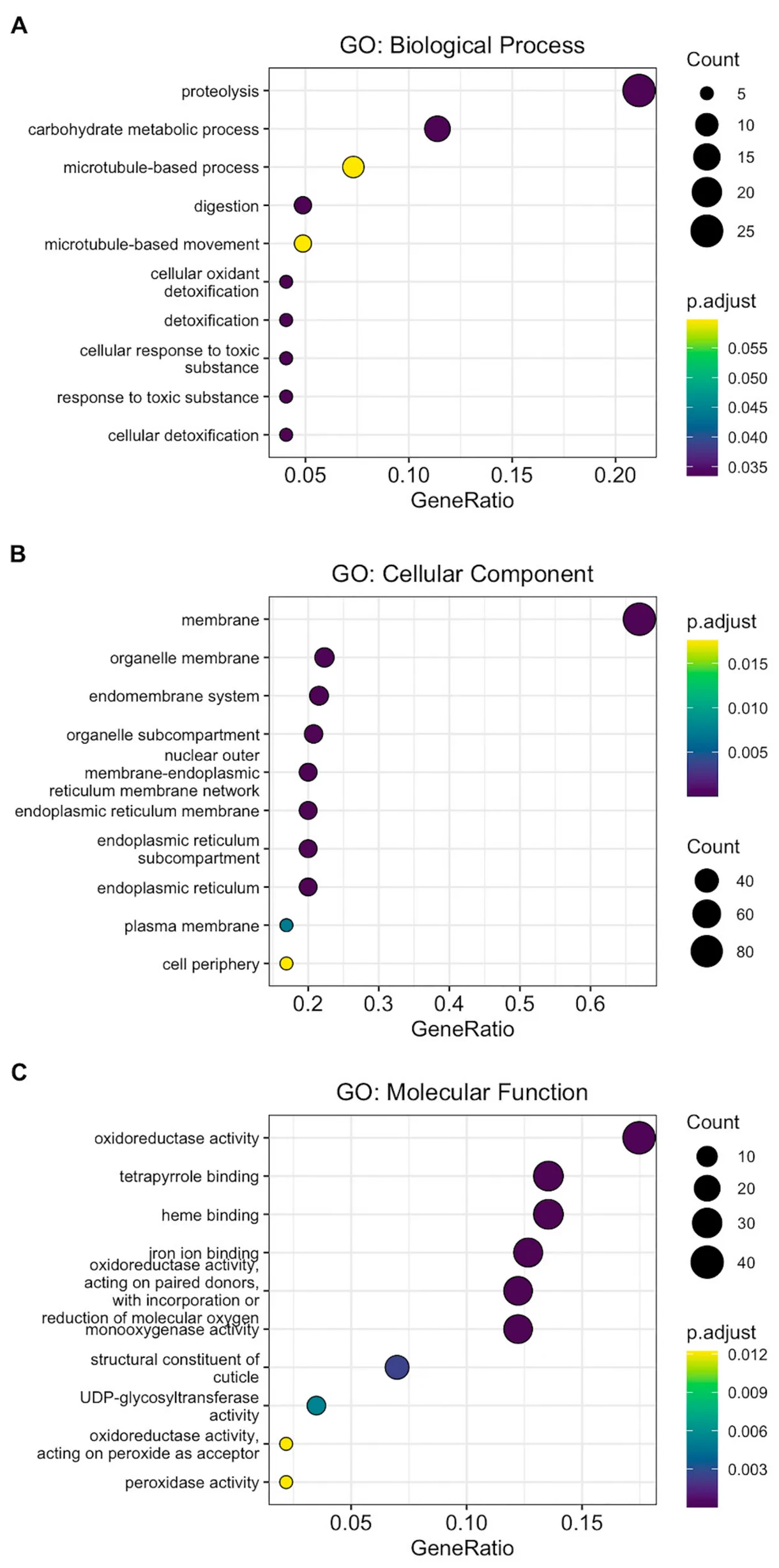
Gene function enrichment analysis of upregulated DEGs in *Ae. aegypti* Field-G2 population highlighting the top 10 enriched Gene Ontology (GO) Biological Processes (BP) terms (**A**), GO Cellular Component (CC) terms (**B**) and GO Molecular Function (MF) terms (**C**).

For the Field-G2 population, enrichment of GO:BP terms shifted toward “proteolysis”, “carbohydrate metabolic process”, and “microtubule-based process”, which may indicate a cellular stress response in *Ae. aegypti* following insecticide exposure (Figure 4A). Insecticide-induced stress can disrupt cellular proteostasis, leading to protein oxidation, misfolding, or damage. To mitigate this, cells activate protein degradation pathways, including upregulation of proteasomes via stress-inducible proteasome chaperones [76]. Furthermore, both stress responses and insecticide detoxification are metabolically demanding processes, necessitating increased carbohydrate metabolism to meet heightened energy requirements. These enriched terms suggest that the transcriptional focus in this population may have shifted from detoxification to stress and energy-related processes, possibly reflecting fitness and energetic trade-offs following repeated sublethal exposures. In addition to enrichment associated with target-site insensitivity, the GO:CC terms “endomembrane system” and “endoplasmic reticulum (ER) membrane” are also enriched in the Field-G2 population (Figure 4B), possibly further supporting the involvement of metabolic detoxification mechanisms in *Ae. aegypti*. CYPs responsible for the oxidative metabolism of pyrethroids are localized in the ER membrane, where xenobiotic detoxification occurs [77,78]. Beyond CYPs, other key detoxification enzymes, including GSTs and CCEs, as well as associated enzymes such as UDPGTs and ABC transporters, are also predominantly ER-resident [59]. This enrichment pattern may highlight the central role of the ER in coordinating metabolic responses to insecticide exposure. Similar to the Field-G0 population, enriched GO:MF terms in the Field-G2 population also include “oxidoreductase activity”, “tetrapyrrole binding”, “heme binding”, and “iron ion binding,” indicating possible consistent involvement of CYP-mediated mechanisms in deltamethrin resistance (Figure 4C). Additionally, both populations show enrichment in genes associated with “UDP-glucuronosyltransferase (UGT) activity” and GO:MF terms related to chitin and cuticle structure. As previously discussed, UGTs contribute to detoxification by facilitating the conjugation and excretion of oxidized insecticide metabolites, while overexpression of chitin and cuticle-associated genes suggests cuticle thickening. This structural modification enhances resistance by reducing insecticide penetration through the exoskeleton [58,70].

**Figure 5:**
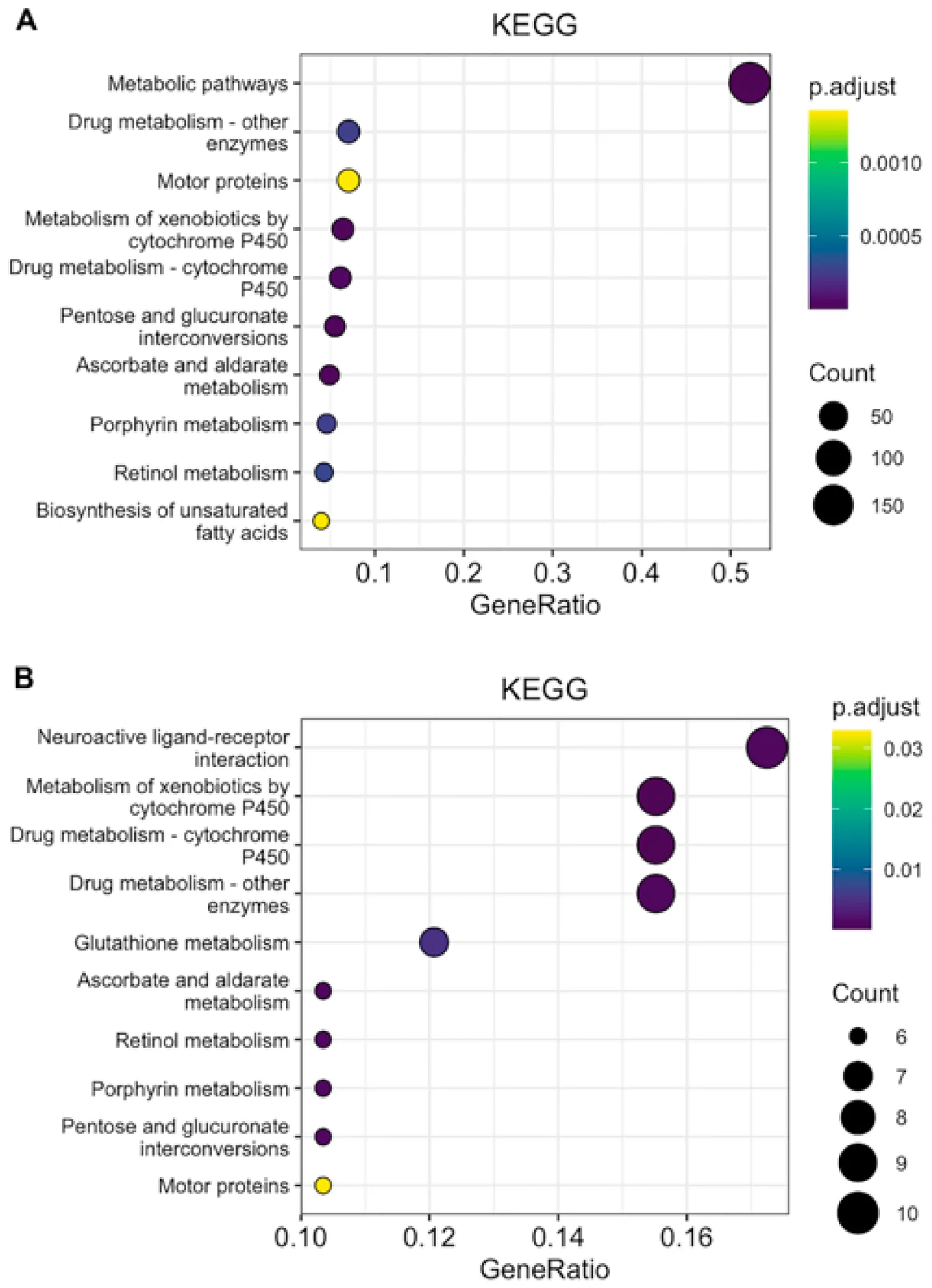
KEGG pathway enrichment analysis of upregulated DEGs in *Ae. aegypti* Field-G0 (**A**) and Field-G2 (**B**) populations showing the top 10 enriched KEGG pathways.

Enriched KEGG pathways, such as “xenobiotic/drug metabolism by cytochrome P450s”, “drug metabolism”, and “metabolism of xenobiotics”, are consistent between the Field-G0 and Field-G2 populations, potentially reinforcing the central role of CYP-mediated detoxification in the metabolic breakdown of deltamethrin (Figure 5). Overall, the results of the GO and KEGG enrichment analyses may supplement the in the presence of established resistance mechanisms in *Ae. aegypti*, particularly highlighting metabolic detoxification mediated by CYPs as a primary mechanism. This pattern is consistently observed in both the Field-G0 and Field-G2 populations.

### 3.5. RNA-Seq Results Validated by Quantitative PCR

Primers were designed for 10 selected DEGs including the overall top three upregulated DEGs (AAEL012664, AAEL004275, and AAEL002138), the overall top three downregulated DEGs (AAEL003297, AAEL013198, and AAEL012565), the top upregulated GST (AAEL001054), and the top three upregulated *CYP*s (AAEL014617, AAEL014613, and AAEL014893) identified in the Field-G2 population (Table S8). Table S9 shows primers’ efficiencies ranging from 84.7% to 112.2%, which are between the acceptable range (80–120%) for reliable qPCR quantification [79,80].

**Figure 6:**
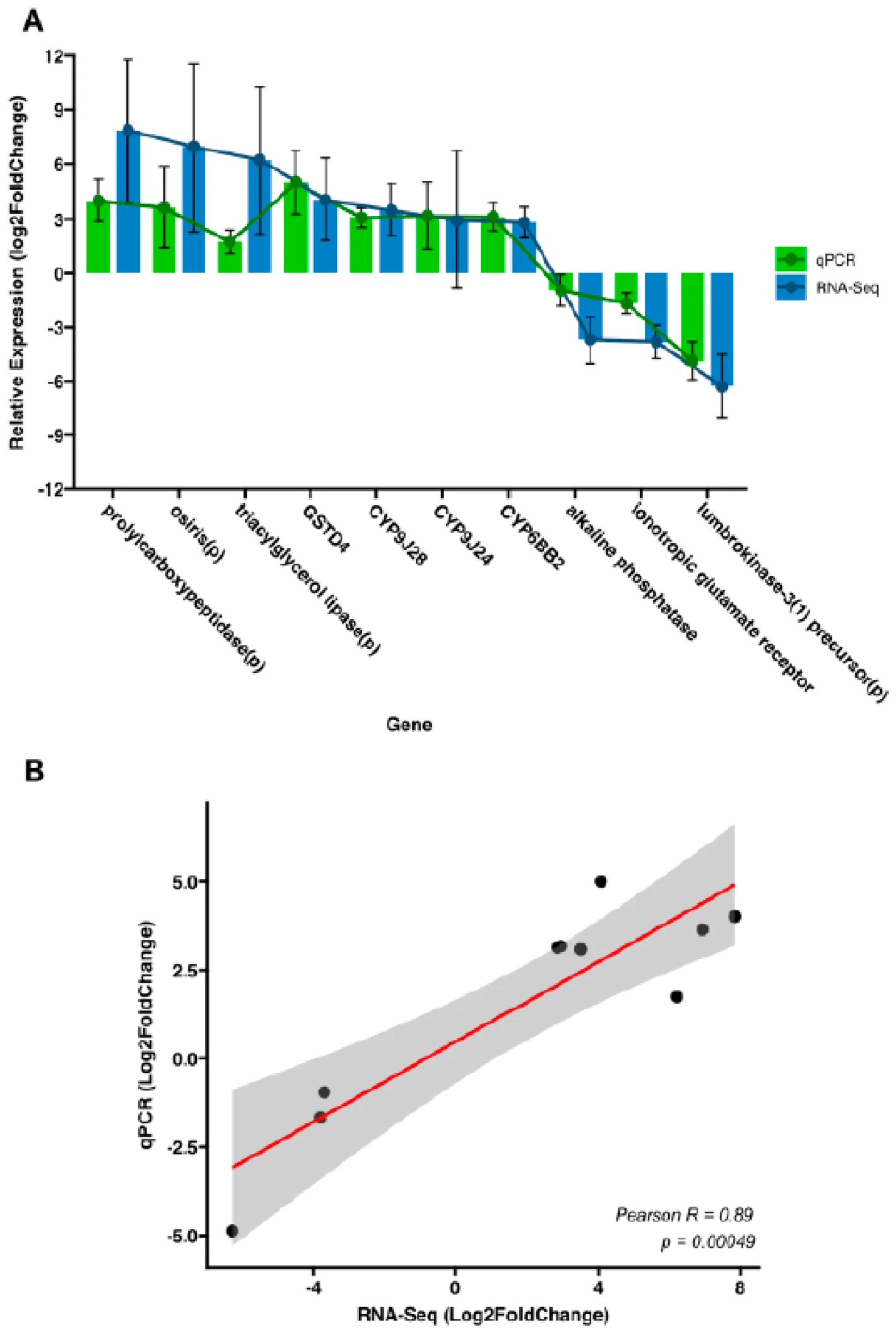
Validation of 10 selected DEGs by quantitative PCR. (**A**) Comparison of the relative expression (log2FC) of the selected DEGs in qPCR (green) and RNA-Seq analysis (DESeq) (blue) [(*p*) = putative]; and (**B**) Pearson correlation analysis of the expression values from qPCR and RNA-Seq. Each DEG is shown as a black dot; the red line represents the regression line (best-fit line), and the shaded area shows the standard error (*p* < 0.05).

Using these primers, normalized expression levels of each selected DEG were assessed in the Field-G2 population relative to the UPLB-UNXP-G122 control population (Figure S4), log2-transformed, and were then compared to their corresponding LFC values obtained from the DGE analysis of the RNA-Seq dataset (Figure 6). Comparison between the two (2) methods shows that qPCR confirms the technical validity of the observed direction of expression changes, both upregulation and downregulation, from the DGE analysis results. The top upregulated genes, including the selected *GST*s and *CYP*s, are consistently validated as upregulated, and the same trend holds true for the downregulated genes. However, as shown by the trend lines in Figure 5, discrepancies in the magnitude of fold changes are observed between the two (2) methods, particularly among the upregulated DEGs. These minor variations are expected, as qPCR and RNA-Seq employ different quantification methods, sensitivity thresholds, and normalization strategies [81]. Moreover, biological variability from the use of field-collected mosquitoes, reflected by the wide error bars, can significantly influence RNA-Seq results [82].

Nevertheless, the overlap of error bars between qPCR and RNA-Seq data, along with consistency in the direction of gene expression changes, indicates concordance in the expression of the selected genes between the two (2) methods, supporting the findings of the RNA-Seq analysis. Furthermore, analysis of fold expression changes in each selected DEG using Pearson correlation revealed a strong (R > 0.80) and statistically significant (*p* < 0.0005) positive correlation (Pearson R = 0.89). This concordance further supports the reliability of the observed gene expression levels from the RNA-Seq analysis.

## 4. Conclusions

Deeper understanding of the molecular mechanisms underlying insecticide resistance is critical for improving vector control strategies. Overall, this study may potentially provide evidence of deltamethrin resistance in *Ae. aegypti* populations from two (2) cities in the Philippines, demonstrated at both the phenotypic and molecular levels. This resistance may possibly be driven by the overexpression of *CYP* genes, as well as *GST* and *CCE* genes, which enhance the metabolic detoxification of pyrethroids, thereby reducing the effectiveness of these insecticides. However, as mentioned previously, the differential gene expressions may also be influenced by different genetic backgrounds and adaptation between the field populations used and the control population, UPLB-UNXP-G122. Nevertheless, these results may inform future resistance surveillance, monitoring programs, and the refinement of current vector control strategies in Pasig City and Parañaque City, Metro Manila, Philippines.

## Figures and Tables

**Table 1:** Assessment for phenotypic resistance frequency based on mortality rates from the WHO susceptibility tube assay [25].

Mortality Rate	Assessment
≥98%	Susceptible
90–97%	Possible resistance that needs confirmation (repeat bioassay)
<90%	Confirmed Resistance

**Table 2:** Summary of CDC CONUS bottle bioassay results for PG13 and PE07 *Aedes aegypti* populations’ susceptibility to deltamethrin at the 30 min diagnostic time after exposure.

Population	Control (Ethanol)			Deltamethrin-Treated (0.75 µg/mL in EtOH)			Resistance Status ^1^
	Alive	Dead	%Mortality	Alive	Dead	% Mortality	
PG13	35	0	0	104	2	1.92	Resistant
PE07	48	0	0	87	3	3.45	Resistant

^1^ based on WHO phenotypic resistance frequency assessment guide (Table 1) [25].

**Table 3:** Summary of WHO tube bioassay results for PG13 and PE07 *Ae. aegypti* populations’ susceptibility to deltamethrin after the 24 h recovery period.

Population	Control (Ethanol)			Deltamethrin-Treated (0.03% in Silicone Oil)			Resistance Status ^1^
	Alive	Dead	%Mortality	Alive	Dead	% Mortality	
PG13	42	0	0	102	3	2.94	Resistant
PE07	65	0	0	112	7	6.25	Resistant

^1^ based on WHO phenotypic resistance frequency assessment guide (Table 1) [25].

**Table 4:** Comparison of LC_50_ of PG13 and PE07 *Ae. aegypti* populations before (G0) and after (G2) two (2) generations of rearing with sublethal exposure (LC_25_) to deltamethrin.

Population	PG13		PE07	
Generation	G0	G2 *	G0	G2 *
LC_50_ (µg/mL)	1.26	2.21	2.15	2.08
CI (95%) ^1^	0.077–5025	4.68–254	6.72–168	3.83–275
Slope ± SE ^2^	1.52 ± 0.4030	1.86 ± 0.5122	1.22 ± 0.5003	1.72 ± 0.233
Goodness-of-fit (R^2^)	0.9468	0.9471	0.8228	0.9914

^1^ confidence interval 95% computed using BioRssay (version 1.10) package in RStudio (version 2024.12.0+467); ^2^ SE = standard error; * reared with sublethal exposure to deltamethrin (LC_25_).

## Data Availability

All data supporting the results of this study are available in the paper and its Supplementary Information. Sequences have been uploaded to the Sequence Read Archive (SRA) at NCBI under accession number: PRJNA1304288.

## Supplementary Materials

The following supporting information can be downloaded at: https://www.mdpi.com/article/10.3390/genes17091093/s1, Table S1: Concentrations and A260/A280 ratios of crude and DNase-treated total RNA extracted from 12 *Aedes aegypti* samples from PG13, PE07 and UPLB; Figure S1: RNA Integrity Numbers (RIN) of the 12 *Aedes aegypti* RNA samples. Electronic gel electrophoresis performed and analyzed using Agilent 2200 TapeStation System (Agilent, Palo Alto, CA, USA); Table S2: Output and quality results of paired-end RNA sequencing of *Aedes aegypti* samples using the NovaSeqTM 6000 Sequencing System (Illumina, CA, USA); Table S3: Percentages of each sample’s reads that aligned and uniquely mapped to the reference genome of *Aedes aegypti* (GCA_002204515.1); Figure S2: Percentage of assigned and unassigned reads per sample; Figure S3: Normalized read coverage profiles along genes (gene bodies); Table S4: List of significantly upregulated annotated genes in Field-G0 samples relative to UPLB-UNXP-G122 samples from DESeq2 v.1.46.0 in R Studio v.2024.12.0+467 (log2FC >1; FDR < 0.1); Table S5: List of significantly downregulated annotated genes in field-G0 samples relative to UPLB-UNXP-G122 samples from DESeq2 v.1.46.0 in R Studio v.2024.12.0+467; Table S6: List of significantly upregulated annotated genes in Field-G2 samples relative to UPLB-UNXP-G122 samples from DESeq2 v.1.46.0 in R Studio v.2024.12.0+467; Table S7: List of significantly downregulated annotated genes in Field-G2 samples relative to UPLB-UNXP-G122 samples from DESeq2 v.1.46.0 in R Studio v.2024.12.0+467; Table S8: Primer sequences and their thermodynamic parameters designed for the validation of selected DEGs; Table S9: qPCR standardization results of each primer pair for gene expression analysis of selected DEGs; Figure S4: qPCR results from relative gene expression analysis of selected DEGs.

## Abbreviations

The following abbreviations are used in this manuscript:

ACL-4	Arthropod Containment Level 4
BSL-2	Biosafety Level 2
LC_25_	Lethal Concentration 25
LC_50_	Lethal Concentration 50 or median lethal concentration
RIN	RNA Integrity Number
DEG	Differentially Expressed Gene
LFC	Log Fold Change
qPCR	Quantitative Polymerase Chain Reaction
CYP	Cytochrome P450 monooxygenase
Cq	Quantification Cycle
