# Supplementary material for: Transcriptome Analysis of Deltamethrin Resistance Mechanisms in Aedes aegypti Populations from Metro Manila, Philippines
Source: Genes (Basel). 2026 Sep 10;17(9):1093. doi: 10.3390/genes17091093 (PMC13606281; doi:10.3390/genes17091093)
Supplement: Supplementary file 1 [file genes-17-01093-s001.zip › Figure_S1.pdf]

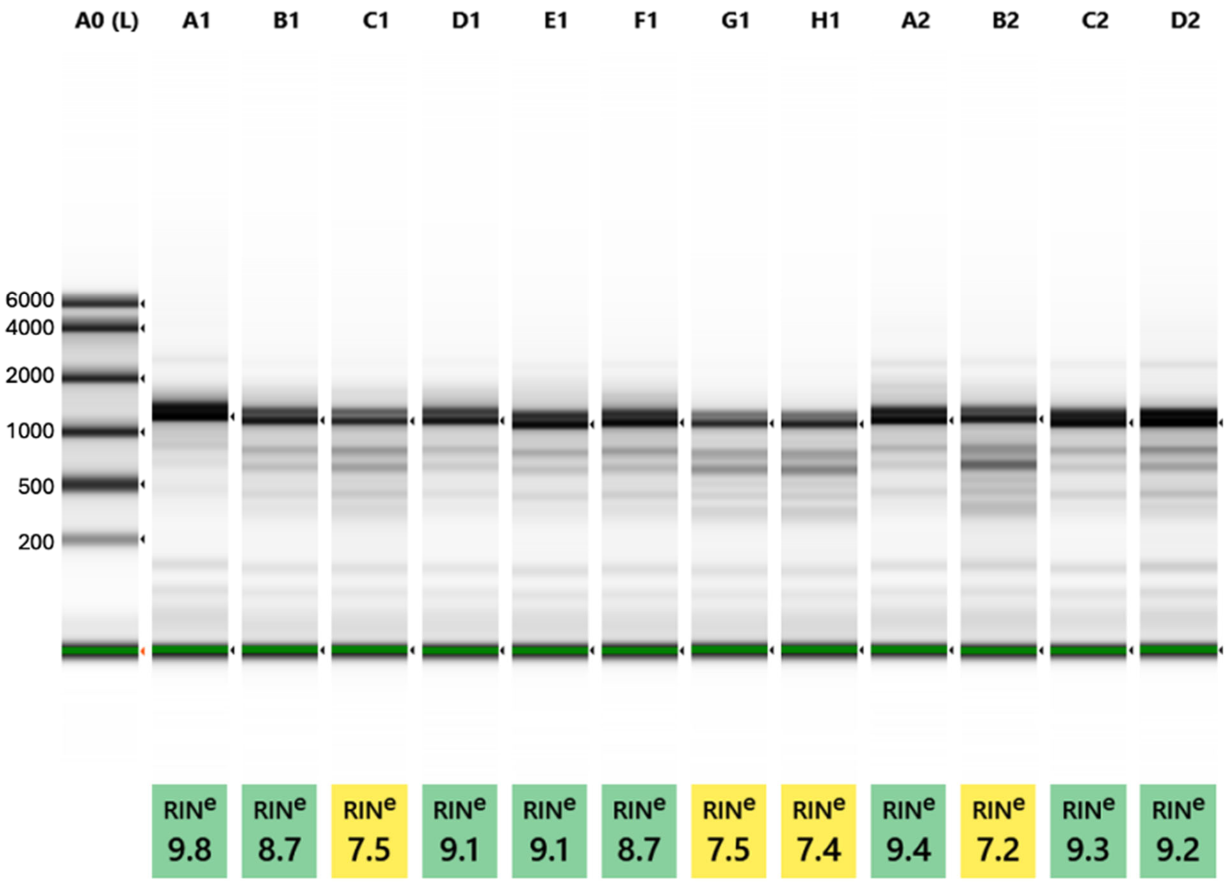

Figure S1. **RNA Integrity Numbers (RIN) of the 12 *Aedes aegypti* RNA samples.** Electronic gel electrophoresis performed and analyzed using Agilent 2200 TapeStation System (Agilent, Palo Alto, CA, USA) (A0 (L): Electronic Ladder; A1: PG13-EXP-G2-1; B1: PG13-EXP-G2-2; C1: PG13-EXP-G2-3; D1: PE07-EXP-G2-1; E1: PE07-EXP-G2-2; F1: PE07-EXP-G2-3; G1: PG13-UNXP-G0-1; H1: PG13-UNXP-G0-2; A2: PE07-UNXP-G0-1; B2: PE07-UNXP-G0-1; C2: UPLB-UNXP-G122-1; D2: UPLB-UNXP-G122-2).
