# Supplementary material for: Transcriptome Analysis of Deltamethrin Resistance Mechanisms in Aedes aegypti Populations from Metro Manila, Philippines
Source: Genes (Basel). 2026 Sep 10;17(9):1093. doi: 10.3390/genes17091093 (PMC13606281; doi:10.3390/genes17091093)
Supplement: Supplementary file 1 [file genes-17-01093-s001.zip › Table_S9.pdf]

Table S9. qPCR standardization results of each primer pair for gene expression analysis of selected DEGs

DEG Primer Pair	Efficiency	R^2	Slope
AAEL012664	85.4%	0.980	-3.730
AAEL004275	105.2%	0.980	-3.203
AAEL002138	94.6%	0.980	-3.459
GSTD4	97.5%	0.999	-3.384
CYP9J28	84.7%	0.981	-3.753
CYP9J24	92.0%	0.993	-3.531
CYP6BB2	91.1%	0.999	-3.556
AAEL003297	112.2%	0.992	-3.060
AAEL013198	105.2%	0.980	-3.203
AAEL0012565	119.9%	0.994	-2.922
RP49*	93.4%	0.998	-3.491

*housekeeping gene (internal control)
