# Supplementary material for: Transcriptome Analysis of Deltamethrin Resistance Mechanisms in Aedes aegypti Populations from Metro Manila, Philippines
Source: Genes (Basel). 2026 Sep 10;17(9):1093. doi: 10.3390/genes17091093 (PMC13606281; doi:10.3390/genes17091093)
Supplement: Supplementary file 1 [file genes-17-01093-s001.zip › Table_S4-S7.pdf]

Table S4. List of significantly upregulated annotated genes in Field-G0 samples relative to UPLB-UNXP-G122 samples from DESeq2 v.1.46.0 in R Studio v.2024.12.0+467 (log2FC >1; FDR < 0.1).

Gene ID	Gene Name	Description	log2FC	p-adj*
AAEL007602	AAEL007602	trypsin, putative	8.7306	0.0002
AAEL025608	AAEL025608	Putative cuticle protein [Source:UniProtKB/TrEMBL;Acc:A0A0P6IXJ4]	8.4379	7.7E-12
AAEL015573	AAEL015573	glycoside hydrolases	7.3352	0.0002
AAEL003881	AAEL003881	ubiquitin, putative	7.2886	2.5E-23
AAEL002138	AAEL002138	triacylglycerol lipase, putative	7.1983	0.0003
AAEL009239	AAEL009239	venom allergen	7.1076	0.0001
AAEL003049	AAEL003049	pupal cuticle protein 78E, putative	6.5737	8.2E-09
AAEL011444	AAEL011444	pupal cuticle protein, putative	6.3356	0.0005
AAEL013512	AAEL013512	pupal cuticle protein, putative	6.2581	0.0021
AAEL003012	AAEL003012	protease m1 zinc metalloprotease	5.6605	1.6E-16
AAEL002241	AAEL002241	cuticle protein, putative	5.5100	0.0050
GSTD4	GSTD4	glutathione S-transferase (GSTD4)	5.3683	1.7E-05
AAEL009556	AAEL009556	Niemann-Pick Type C-2, putative	5.1126	0.0015
AAEL009801	AAEL009801	cuticle protein, putative	5.0292	0.0002
AAEL009566	AAEL009566	apolipoprotein D, putative	5.0267	0.0003
AAEL003259	AAEL003259	pupal cuticle protein 78E, putative	4.9178	0.0001
AAEL015163	AAEL015163	cuticle protein, putative	4.8621	2.8E-10
AAEL010134	AAEL010134	Pupal cuticle protein, putative [Source:UniProtKB/TrEMBL;Acc:A0A1S4G2T5]	4.8163	0.0011
AAEL001951	Act-4	actin	4.8037	5.0E-05
AAEL011169	AAEL011169	hexamerin 2 beta	4.5964	9.4E-15
AAEL014617	CYP9J28	cytochrome P450	4.5641	0.0002
AAEL004275	AAEL004275	Osiris, putative [Source:UniProtKB/TrEMBL;Acc:A0A1S4F763]	4.4463	0.0161
AAEL002092	AAEL002092	cuticle protein, putative	4.3226	0.0001

Gene ID	Gene Name	Description	log2FC	p-adj*
AAEL009628	AAEL009628	short-chain dehydrogenase	4.1769	0.0005
AAEL011375	AAEL011375	trypsin, putative	4.0606	0.0045
AAEL009451	AAEL009451	actin	4.0505	0.0004
AAEL009904	AAEL009904	PDZ domain-containing protein [Source:UniProtKB/TrEMBL;Acc:A0A1S4FP41]	4.0504	0.0001
AAEL014371	AAEL014371	glucosyl/glucuronosyl transferases	4.0285	0.0002
AAEL001301	AAEL001301	CRAL-TRIO domain-containing protein [Source:UniProtKB/TrEMBL;Acc:A0A1S4EYD7]	3.8871	0.0005
AAEL013283	AAEL013283	serine-type enodpeptidase	3.8554	0.0001
AAEL000678	AAEL000678	alpha-amylase	3.8489	0.0015
AAEL012700	AAEL012700	ATP-binding cassette sub-family A member 3, putative	3.8238	0.0001
AAEL008749	AAEL008749	udp-glucuronosyltransferase	3.7671	0.0015
AAEL007281	AAEL007281	stretchin-mlck	3.7399	0.0024
AAEL006919	AAEL006919	serine-type enodpeptidase,	3.7282	6.6E-21
AAEL004003	AAEL004003	glucose dehydrogenase	3.7120	0.0005
AAEL000878	AAEL000878	cuticle protein, putative	3.7066	2.3E-05
AAEL004369	AAEL004369	alpha-glucosidase	3.6843	0.0019
AAEL006327	AAEL006327	sulfotransferase (sult)	3.6799	0.0008
AAEL000651	AAEL000651	alpha-amylase	3.6753	0.0004
AAEL013757	AAEL013757	hexamerin 2 beta	3.6553	3.9E-08
AAEL004103	AAEL004103	deoxyribonuclease I, putative	3.6072	0.0033
AAEL015139	AAEL015139	Niemann-Pick Type C-2, putative	3.5795	0.0188
AAEL011504	AAEL011504	pupal cuticle protein, putative	3.5092	0.0017
AAEL007938	AAEL007938	serine-type enodpeptidase,	3.4654	0.0303
AAEL013853	CTLGA2	C-Type Lectin (CTL) - galactose binding.	3.4653	0.0035
AAEL002185	AAEL002185	cuticle protein, putative	3.4170	6.5E-07

Gene ID	Gene Name	Description	log2FC	p-adj*
AAEL002246	AAEL002246	cuticle protein, putative	3.3828	0.0149
AAEL010894	AAEL010894	carbonic anhydrase II, putative	3.3732	6.1E-05
AAEL014893	CYP6BB2	cytochrome P450	3.3616	0.0008
AAEL001318	AAEL001318	CRAL/TRIO domain-containing protein	3.3564	2.6E-06
AAEL008767	AAEL008767	serine protease	3.3486	4.4E-05
AAEL004897	AAEL004897	brain chitinase and chia	3.3363	0.0008
AAEL012101	AAEL012101	tubulin alpha chain	3.3306	0.0029
AAEL007684	AAEL007684	tektin, putative	3.2957	0.0009
AAEL008045	AAEL008045	hexamerin 2 beta	3.2692	1.0E-04
AAEL005759	AAEL005759	Rhodanese domain-containing protein [Source:UniProtKB/TrEMBL;Acc:A0A1S4FBL6]	3.2595	0.0074
AAEL002313	AAEL002313	glucose transport protein, putative	3.2342	0.0070
AAEL007369	AAEL007369	homeobox protein even skipped	3.2196	0.0043
AAEL000757	AAEL000757	anterior fat body protein	3.1323	0.0072
AAEL008764	AAEL008764	cuticle protein, putative	3.1299	0.0002
AAEL013276	AAEL013276	acid phosphatase	3.1256	5.0E-07
AAEL001745	AAEL001745	candidate tumor suppressor protein	3.0945	0.0083
AAEL001822	AAEL001822	glucosyl/glucuronosyl transferases	3.0781	0.0059
AAEL006179	AAEL006179	tubulin alpha chain	3.0748	0.0003
AAEL002191	AAEL002191	cuticle protein, putative	3.0601	0.0138
AAEL014609	CYP9J26	cytochrome P450	3.0143	0.0034
AAEL007669	AAEL007669	oxidoreductase	3.0121	0.0031
AAEL015637	AAEL015637	serine carboxypeptidase, putative	2.9880	0.0039
AAEL001899	AAEL001899	juvenile hormone-inducible protein, putative	2.9556	0.0001
AAEL004788	AAEL004788	Osiris, putative	2.9487	0.0258
AAEL008285	AAEL008285	pupal cuticle protein, putative	2.9231	0.0056

Gene ID	Gene Name	Description	log2FC	p-adj*
AAEL008006	AAEL008006	3-hydroxyacyl-coa dehyrogenase	2.9084	0.0006
AAEL011822	AAEL011822	gamma glutamyl transpeptidases	2.9079	4.9E-10
AAEL003589	AAEL003589	transcription factor, putative	2.8901	0.0002
AAEL000667	AAEL000667	alpha-amylase	2.8746	0.0040
AAEL015020	AAEL015020	glycoside hydrolases	2.8527	0.0018
AAEL013385	AAEL013385	brain chitinase and chia	2.8502	0.0001
AAEL014613	CYP9J24	cytochrome P450	2.8499	0.0002
AAEL008218	AAEL008218	serine-type enodpeptidase,	2.8463	0.0293
AAEL007582	AAEL007582	granzyme A precursor, putative	2.8409	0.0004
AAEL008066	AAEL008066	trkB protein, putative	2.7970	0.0045
AAEL012762	CYP325N2	cytochrome P450	2.7967	0.0058
AAEL008782	AAEL008782	serine-type enodpeptidase,	2.7912	0.0007
AAEL009124	CYP6N12	cytochrome P450	2.7432	0.0015
AAEL010396	AAEL010396	secreted ferritin G subunit precursor, putative	2.7251	0.0033
AAEL002718	AAEL002718	chitin synthase	2.7210	0.0079
AAEL009561	AAEL009561	apolipoprotein D, putative	2.7194	0.0036
AAEL006797	AAEL006797	F-box/leucine rich repeat protein	2.7095	0.0019
AAEL002229	AAEL002229	cuticle protein, putative	2.6784	0.0208
AAEL007432	AAEL007432	serine collagenase 1 precursor, putative	2.6663	0.0122
AAEL006206	AAEL006206	N-twist [Source:UniProtKB/TrEMBL;Acc:A0A1S4FD16]	2.6586	0.0015
AAEL006691	AAEL006691	fibrinogen and fibronectin	2.6557	0.0005
AAEL000057	TOLL5B	Toll-like receptor	2.6540	0.0023
AAEL000762	LRIM19	leucine-rich immune protein (Coil-less)	2.6157	5.0E-05
AAEL005906	AAEL005906	serine protease	2.6112	5.3E-06
AAEL006353	AAEL006353	sulfotransferase (sult)	2.6058	0.0026
AAEL006702	AAEL006702	fibrinogen and fibronectin	2.5925	6.0E-06

Gene ID	Gene Name	Description	log2FC	p-adj*
AAEL000382	AAEL000382	myosin motor, putative	2.5871	0.0042
AAEL004580	AAEL004580	beta-galactosidase	2.5632	0.0100
AAEL005138	AAEL005138	glucosyl/glucuronosyl transferases	2.5351	0.0014
AAEL004674	AAEL004674	pupal cuticle protein 78E, putative	2.5223	0.0049
AAEL004778	AAEL004778	acyl-coa dehydrogenase	2.5214	0.0034
AAEL002909	AAEL002909	lysosomal acid lipase, putative	2.5200	0.0051
AAEL005188	AAEL005188	alpha-galactosidase/alpha-n-acetylgalactosaminidase	2.5100	0.0053
AAEL009680	AAEL009680	chymotrypsin, putative	2.5096	0.0090
AAEL005386	AAEL005386	collagen alpha chain, anopheles	2.4968	0.0036
AAEL018028	CYP325Y3	Cytochrome P450 [Source:UniProtKB/TrEMBL;Acc:A0A1S4G6V3]	2.4829	0.0030
AAEL007063	AAEL007063	lipase	2.4797	0.0078
AAEL004067	AAEL004067	mitochondrial benzodiazepine receptor, putative	2.4779	0.0117
AAEL002039	AAEL002039	NADPH oxidase	2.4777	0.00429019
AAEL002309	TPX4	Thioredoxin Peroxidase.	2.4723	0.00059113
AAEL000250	AAEL000250	forkhead box protein (AaegFOXP)	2.4705	5.94E-05
AAEL008456	AAEL008456	alpha-amylase	2.4692	0.0043576
AAEL003294	AAEL003294	fibrinogen and fibronectin	2.4654	0.00012011
AAEL014567	AAEL014567	oviductin	2.4636	1.59E-06
AAEL017460	AAEL017460	Angiotensin-converting enzyme [Source:UniProtKB/TrEMBL;Acc:A0A1S4G6D0]	2.4511	0.0011377
AAEL005969	AAEL005969	phospholipase b, plb1	2.4284	0.01710716
AAEL013218	AAEL013218	thiamine transporter	2.4271	0.00258727
AAEL012636	AAEL012636	cytochrome b5, putative	2.4222	5.57E-08
AAEL013722	AAEL013722	partner of burs, putative	2.4121	0.01363114
AAEL010681	AAEL010681	sodium/chloride dependent neurotransmitter transporter	2.4050	0.00015649

Gene ID	Gene Name	Description	log2FC	p-adj*
AAEL009505	AAEL009505	Ig-like domain-containing protein [Source:UniProtKB/TrEMBL;Acc:A0A1S4FMI3]	2.3859	0.00398898
AAEL009748	AAEL009748	2-hydroxyphytanoyl-coa lyase [Source:UniProtKB/TrEMBL;Acc:Q16UY3]	2.3620	2.61E-06
AAEL006703	AAEL006703	lumbrokinase-3(1) precursor, putative	2.3599	0.01112475
AAEL009338	CTL10	C-Type Lectin (CTL).	2.3502	0.00652116
AAEL014432	AAEL014432	fibrinogen and fibronectin	2.3445	0.04606425
AAEL013417	AAEL013417	fibrinogen and fibronectin	2.3302	0.02354781
AAEL007559	AAEL007559	guanyl-nucleotide exchange factor, putative	2.3142	7.86E-06
AAEL008844	AAEL008844	calcium-binding protein, putative	2.3017	9.67E-07
AAEL005026	AAEL005026	ATP-dependent bile acid permease	2.2959	7.62E-06
AAEL022646	AAEL022646	Larval chymotrypsin-like protein [Source:UniProtKB/TrEMBL;Acc:Q8T639]	2.2845	0.01529816
AAEL000797	AAEL000797	dimethylaniline monooxygenase	2.2844	0.00363491
AAEL013906	AAEL013906	bone morphogenetic protein 5/7, bmp5/7	2.2843	0.00384603
AAEL015631	AAEL015631	asparagine synthetase	2.2820	3.24E-08
AAEL003954	AAEL003954	juvenile hormone-inducible protein, putative	2.2795	0.00020493
AAEL005621	GPROP4	long wavelength sensitive opsin	2.2767	0.00131371
AAEL001312	CYP9M6	Cytochrome P450 monooxygenase CYP9M6v2 [Source:UniProtKB/TrEMBL;Acc:X5HYQ8]	2.2749	0.00138891
AAEL012268	AAEL012268	brain chitinase and chia	2.2643	0.02217506
AAEL011981	AAEL011981	glutamate decarboxylase	2.2568	5.90E-05
AAEL014978	AAEL014978	adult cuticle protein, putative	2.2543	0.01134845
AAEL014437	AAEL014437	CHK domain-containing protein [Source:UniProtKB/TrEMBL;Acc:A0A1S4G1W8]	2.2376	0.0028525
AAEL014527	AAEL014527	potassium-dependent sodium-calcium exchanger, putative	2.2364	8.19E-05
AAEL010157	AAEL010157	microsomal glutathione s-transferase	2.2283	0.01564184

Gene ID	Gene Name	Description	log2FC	p-adj*
AAEL002669	AAEL002669	AMP dependent ligase	2.2276	2.72E-05
AAEL011809	AAEL011809	glucose dehydrogenase	2.2263	0.00713828
AAEL009881	AAEL009881	dynein heavy chain	2.2195	0.00046646
AAEL000012	Gr6	gustatory receptor Gr6	2.2147	0.00553318
AAEL008289	AAEL008289	pupal cuticle protein 78E, putative	2.2142	0.00788881
AAEL007424	AAEL007424	phospholipid scramblase, putative	2.2055	0.02489131
AAEL014438	AAEL014438	juvenile hormone-inducible protein, putative	2.2014	0.0136008
AAEL009214	AAEL009214	diazepam binding inhibitor, putative	2.2010	0.01134984
AAEL002165	AAEL002165	Putative hydroxypyruvate isomerase [Source:UniProtKB/TrEMBL;Acc:A0A1S4F0V8]	2.1986	0.00115248
AAEL002692	AAEL002692	Beat protein [Source:UniProtKB/TrEMBL;Acc:A0A1S4F2K6]	2.1870	1.91E-05
AAEL010171	PGRPLB	peptidoglycan recognition protein (Long)	2.1772	0.00178893
AAEL009951	AAEL009951	dimeric dihydrodiol dehydrogenase	2.1733	0.02165934
AAEL025597	AAEL025597	Amino acid transporter [Source:UniProtKB/TrEMBL;Acc:A0A1S4FFR9]	2.1670	0.02137915
AAEL013829	AAEL013829	cuticle protein, putative	2.1622	0.01311705
AAEL000690	AAEL000690	steroid dehydrogenase	2.1528	0.00312882
AAEL010570	AAEL010570	acetylcholine receptor, beta-type subunit invertebrate	2.1518	0.00013653
AAEL000341	AAEL000341	basic helix-loop-helix protein	2.1467	0.00016767
AAEL008660	AAEL008660	Fig-alpha [Source:UniProtKB/TrEMBL;Acc:A0A1S4FK79]	2.1458	0.00482309
AAEL014614	AAEL014614	cytochrome P450	2.1376	0.00676112
AAEL006961	AAEL006961	lipase	2.1335	0.02164978
AAEL004273	AAEL004273	short-chain dehydrogenase	2.1332	0.01375048
AAEL014846	inx1	Innexin [Source:UniProtKB/TrEMBL;Acc:Q16FA3]	2.1278	0.00553768
AAEL011263	AAEL011263	phosphatidylethanolamine-binding protein	2.1247	0.00012665

Gene ID	Gene Name	Description	log2FC	p-adj*
AAEL014297	AAEL014297	xenotropic and polytropic murine leukemia virus receptor xpr1	2.1232	0.00151978
AAEL010386	AAEL010386	glucosyl/glucuronosyl transferases	2.1101	0.01526945
AAEL005772	OBP22	odorant binding protein OBP22	2.1074	0.00961001
AAEL008587	AAEL008587	glutamate receptor, ionotropic, N-methyl d-aspartate	2.1073	7.48E-08
AAEL002716	AAEL002716	Arrestin_C domain-containing protein [Source:UniProtKB/TrEMBL;Acc:A0A1S4F2U1]	2.1042	0.00442594
AAEL002354	HPX5	heme peroxidase	2.1042	0.01791292
AAEL000254	AAEL000254	nuclear factor i	2.1009	0.00141738
AAEL002192	AAEL002192	M12 mutant protein precursor, putative	2.0951	0.00150741
AAEL003123	AAEL003123	deoxyribonuclease I, putative	2.0913	0.00527601
AAEL002547	AAEL002547	dipeptidase	2.0901	1.85E-05
AAEL013802	AAEL013802	Fatty acyl-CoA reductase [Source:UniProtKB/TrEMBL;Acc:A0A1S4FZZ5]	2.0852	0.0333002
AAEL014520	AAEL014520	follistatin	2.0827	8.83E-05
AAEL014457	AAEL014457	neuronal cell adhesion molecule	2.0818	5.57E-08
AAEL002088	AAEL002088	sodium channel, auxiliary protein, putative	2.0749	0.00011532
AAEL012776	AAEL012776	protease m1 zinc metalloprotease	2.0669	0.01005322
AAEL010382	AAEL010382	aldehyde oxidase	2.0610	0.01877416
AAEL000075	Gr9	gustatory receptor Gr9	2.0554	0.02225925
AAEL000556	CTL25	C-Type Lectin (CTL25)	2.0496	8.89E-11
AAEL014246	AAEL014246	glucosyl/glucuronosyl transferases	2.0386	0.00015617
AAEL009193	AAEL009193	glucose dehydrogenase	2.0318	0.03026123
AAEL007473	CYP6AH1	cytochrome P450	2.0317	0.01954445
AAEL006699	AAEL006699	fibrinogen and fibronectin	2.0285	0.00240322
AAEL003091	AAEL003091	glucosyl/glucuronosyl transferases	2.0274	0.01526945
AAEL006784	CYP9J17	cytochrome P450	2.0272	0.02193796

Gene ID	Gene Name	Description	log2FC	p-adj*
AAEL012356	GPRSMS	GPCR Somatostatin Family	2.0250	0.00125904
AAEL014541	AAEL014541	maltose phosphorylase	2.0239	0.01373007
AAEL006982	AAEL006982	lipase	2.0220	0.0067523
AAEL007700	AAEL007700	ERm	2.0186	0.03202179
AAEL012349	AAEL012349	lipase 1 precursor	2.0137	2.50E-05
AAEL000505	AAEL000505	CHK domain-containing protein [Source:UniProtKB/TrEMBL;Acc:A0A1S4EW44]	2.0122	0.02180581
AAEL010783	AAEL010783	sodium/potassium-dependent ATPase beta-2 subunit	2.0062	5.49E-05
AAEL005581	AAEL005581	norepinephrine/norepinephrine transporter	1.9939	6.09E-05
AAEL002972	AAEL002972	brain chitinase and chia	1.9890	0.02691331
AAEL007194	AAEL007194	pupal cuticle protein, putative	1.9853	0.02778018
AAEL008641	AAEL008641	GTP-binding protein (o) alpha subunit, gnao	1.9851	0.00036725
AAEL005363	AAEL005363	galactosyltransferase	1.9797	0.00086281
AAEL006487	AAEL006487	sodium/solute symporter	1.9774	2.64E-05
AAEL010478	AAEL010478	sugar transporter	1.9747	0.01711515
AAEL002485	WDY	WD repeat-containing protein on Y chromosome (WD40 Y)	1.9675	0.03346418
AAEL000515	AAEL000515	juvenile hormone-inducible protein, putative	1.9646	0.01347885
AAEL014412	CYP304B2	cytochrome P450	1.9606	0.00761131
AAEL005803	AAEL005803	Gastrin-releasing peptide receptor [Source:UniProtKB/TrEMBL;Acc:A0A411JKA4]	1.9543	0.00519572
AAEL005076	AAEL005076	alanyl aminopeptidase	1.9525	1.23E-05
AAEL001548	AAEL001548	glucosyl/glucuronosyl transferases	1.9500	0.01192506
AAEL010102	AAEL010102	tetraspanin, putative	1.9498	0.00308878
AAEL014853	AAEL014853	otoferlin	1.9472	0.00015617
AAEL003079	AAEL003079	glucosyl/glucuronosyl transferases	1.9407	0.02995257

Gene ID	Gene Name	Description	log2FC	p-adj*
AAEL003894	GNBPB5	Gram-Negative Binding Protein (GNBP) or Beta-1 3-Glucan Binding Protein (BGBP).	1.9397	7.41E-05
AAEL007942	AAEL007942	fibrinogen and fibronectin	1.9353	0.00423576
AAEL008003	AAEL008003	serine protease	1.9349	0.00398898
AAEL012206	AAEL012206	microtubule-associated protein tau	1.9327	0.00067111
AAEL007673	AAEL007673	alpha-amylase	1.9224	0.03056933
AAEL008751	AAEL008751	glucosyl/glucuronosyl transferases	1.9221	0.03421494
AAEL001687	AAEL001687	adenylate cyclase	1.9220	0.03191968
AAEL004582	AAEL004582	beta-galactosidase	1.9195	0.02892855
AAEL010582	GSTD11	glutathione transferase	1.9183	0.02840693
AAEL007518	AAEL007518	complexin, putative	1.9151	0.00011456
AAEL012868	AAEL012868	cmp-n-acetylneuraminic acid synthase	1.9140	0.00398898
AAEL001139	AAEL001139	br serine/threonine-protein kinase	1.9133	6.70E-05
AAEL005353	AAEL005353	Sugar transporter SWEET [Source:UniProtKB/TrEMBL;Acc:Q17A97]	1.9005	0.00254836
AAEL001140	AAEL001140	MutS protein homolog 4	1.8999	1.09E-06
AAEL022538	AAEL022538	Putative bisphosphate 3'-nucleotidase bpnt1/inositol polyphosphate 1-phosphatase [Source:UniProtKB/TrEMBL;Acc:A0A0P6IYC3]	1.8915	0.00153917
AAEL012248	AAEL012248	Histamine-gated chloride channel subunit [Source:UniProtKB/TrEMBL;Acc:A0A1S4FVK1]	1.8898	0.00046062
AAEL002827	AAEL002827	ATP synthase beta subunit	1.8893	0.00199258
AAEL002917	AAEL002917	CHK domain-containing protein [Source:UniProtKB/TrEMBL;Acc:A0A1S4F357]	1.8889	0.00017117
AAEL005072	AAEL005072	MRAS2, putative	1.8878	0.00029198
AAEL011882	AAEL011882	trypsin-zeta, putative	1.8859	0.00012361
AAEL008036	AAEL008036	dynein heavy chain	1.8855	0.00091236
AAEL010395	AAEL010395	beat protein	1.8823	0.00040939
AAEL012691	AAEL012691	sodium/solute symporter	1.8792	0.00182393

Gene ID	Gene Name	Description	log2FC	p-adj*
AAEL001696	AAEL001696	paired box protein, putative	1.8758	0.00094514
AAEL000855	AAEL000855	mannosyltransferase 1,	1.8738	0.01886097
AAEL006704	AAEL006704	fibrinogen and fibronectin	1.8653	0.00759549
AAEL003905	AAEL003905	alkaline phosphatase	1.8606	0.03044938
AAEL006809	AAEL006809	voltage-gated ion channel	1.8561	0.00021203
AAEL006978	AAEL006978	protein-glutamine gamma-glutamyltransferase	1.8557	0.02300859
AAEL007500	AAEL007500	CRAL_TRIO_N domain-containing protein [Source:UniProtKB/TrEMBL;Acc:A0A1S4FGV5]	1.8527	0.01769499
AAEL001835	AAEL001835	sodium/solute symporter	1.8517	0.01231181
AAEL005182	CCEJHE4F	Carboxy/choline esterase	1.8509	0.00035467
AAEL006685	AAEL006685	G-protein, gamma-subunit, putative	1.8460	0.00468307
AAEL009072	AAEL009072	ribonucleoprotein	1.8454	2.64E-05
AAEL002520	AAEL002520	apolipoprotein D, putative	1.8451	0.02137915
AAEL002759	AAEL002759	tropomyosin invertebrate	1.8449	0.00045768
AAEL006932	AAEL006932	transcription factor ap-2	1.8449	1.02E-05
AAEL006334	AAEL006334	sulfotransferase (sult)	1.8434	0.03353504
AAEL013649	AAEL013649	elongase, putative	1.8348	0.03193394
AAEL001878	AAEL001878	lipase	1.8346	0.00642763
AAEL011553	AAEL011553	trypsin	1.8346	0.034563
AAEL004533	GPRMGL2	GPCR Metabotropic glutamate Family	1.8342	0.00040685
AAEL000871	AAEL000871	Fatty acyl-CoA reductase [Source:UniProtKB/TrEMBL;Acc:Q17MY8]	1.8333	0.06573038
AAEL011070	CTLGA3	C-Type Lectin (CTL) - galactose binding.	1.8325	0.01067219
AAEL003504	AAEL003504	emx homeobox protein	1.8309	0.00019988
AAEL002997	CLIPD3	Clip-Domain Serine Protease family D.	1.8286	0.02995257
AAEL005961	AAEL005961	actin	1.8252	0.00010827

Gene ID	Gene Name	Description	log2FC	p-adj*
AAEL011957	AAEL011957	elongase, putative	1.8244	0.02393224
AAEL001068	AAEL001068	myosin light chain 2V, putative	1.8173	0.00229414
AAEL006822	AAEL006822	otopetrin	1.8168	5.82E-05
AAEL004032	AAEL004032	acetylcholine receptor protein alpha 1, 2, 3, 4 invertebrate	1.8162	0.00513639
AAEL013229	AAEL013229	Tubulin alpha chain [Source:UniProtKB/TrEMBL;Acc:Q16JS3]	1.8087	0.00931917
AAEL014258	AAEL014258	monocarboxylate transporter	1.8086	0.00033828
AAEL014344	AAEL014344	adam (a disintegrin and metalloprotease)	1.8061	0.03138135
AAEL011332	AAEL011332	dopamine beta hydroxylase	1.7994	0.00322789
AAEL026044	AAEL026044	Sterol carrier protein 2 [Source:UniProtKB/TrEMBL;Acc:Q86PR3]	1.7992	0.00467848
AAEL004678	AAEL004678	rhomboid	1.7977	0.00050609
AAEL017297	CYP6M9	cytochrome P450	1.7954	0.0167181
AAEL011318	AAEL011318	guanylate cyclase	1.7942	0.00010065
AAEL013496	PPO8	prophenoloxidase	1.7846	4.54E-05
AAEL006601	AAEL006601	50-kda dystrophin-associated glycoprotein, putative	1.7818	0.00159786
AAEL002638	CYP9J6	cytochrome P450	1.7808	0.02195602
AAEL001228	AAEL001228	myelin transcription factor 1, myt1	1.7785	0.00079851
AAEL001517	AAEL001517	carboxylesterase	1.7758	7.36E-08
AAEL005392	AAEL005392	dihydropyridine-sensitive l-type calcium channel	1.7749	0.00227911
AAEL003436	AAEL003436	transcription factor coe3	1.7749	0.00121701
AAEL001658	AAEL001658	sodium-dependent phosphate transporter	1.7694	0.034563
AAEL000842	AAEL000842	microsomal dipeptidase	1.7675	0.03876269
AAEL013611	AAEL013611	AMP dependent ligase	1.7649	6.28E-06
AAEL010120	AAEL010120	chondroitin 4-sulfotransferase	1.7589	0.001102

Gene ID	Gene Name	Description	log2FC	p-adj*
AAEL008410	AAEL008410	helix-loop-helix transcription factor	1.7588	0.00792137
AAEL010712	AAEL010712	low-density lipoprotein receptor (ldl)	1.7585	0.00049078
AAEL012949	AAEL012949	Transferrin [Source:UniProtKB/TrEMBL;Acc:A0A1S4FXI3]	1.7567	0.00520299
AAEL001857	AAEL001857	SET domain-containing protein [Source:UniProtKB/TrEMBL;Acc:A0A1S4EZZ1]	1.7566	0.00038137
AAEL007233	AAEL007233	zinc finger protein	1.7536	0.00109749
AAEL013997	AAEL013997	guanine nucleotide-binding protein beta 3 (g protein beta3)	1.7481	0.00307573
AAEL000146	AAEL000146	nitrilase, putative	1.7457	0.02840911
AAEL006425	AAEL006425	trypsin	1.7452	0.02932602
AAEL013263	AAEL013263	high affinity copper transporter, putative	1.7438	0.00311285
AAEL006921	AAEL006921	calmodulin	1.7435	0.00126509
AAEL013109	AAEL013109	glutamate transporter	1.7411	0.00603429
AAEL006452	AAEL006452	n-acetylgalactosaminyltransferase	1.7398	0.00296681
AAEL013026	AAEL013026	guanylate cyclase	1.7370	0.00190404
AAEL006760	AAEL006760	Bc8 orange interacting protein, putative [Source:UniProtKB/TrEMBL;Acc:A0A1S4FEK1]	1.7348	0.04510651
AAEL014539	AAEL014539	maltose phosphorylase	1.7343	0.00869063
AAEL006526	AAEL006526	neurotransmitter gated ion channel	1.7278	0.01139951
AAEL004522	GAM1	gambicin anti-microbial peptide	1.7271	0.02583655
AAEL006051	AAEL006051	beta-sarcoglycan	1.7223	0.00135548
AAEL005412	AAEL005412	annexin x	1.7216	4.57E-06
AAEL009125	CYP6M10	cytochrome P450	1.7153	0.01530191
AAEL007495	AAEL007495	phosphoglycerate mutase	1.6996	0.00203734
AAEL000595	AAEL000595	beat protein	1.6958	0.00057652
AAEL005130	AAEL005130	zinc finger protein	1.6925	1.71E-05
AAEL011132	AAEL011132	metalloproteinase, putative	1.6904	0.05284766

Gene ID	Gene Name	Description	log2FC	p-adj*
AAEL006522	AAEL006522	turtle protein, isoform	1.6903	0.00029063
AAEL014227	shakB	innexin shaking-B	1.6876	8.39E-05
AAEL009128	CYP6M6	cytochrome P450	1.6876	0.03107126
AAEL011528	AAEL011528	triacylglycerol lipase, putative	1.6866	0.04248602
AAEL009185	AAEL009185	arginine or creatine kinase	1.6865	0.01433127
AAEL008715	AAEL008715	AAA ATPase	1.6862	0.04048665
AAEL006630	AAEL006630	CHK domain-containing protein [Source:UniProtKB/TrEMBL;Acc:A0A1S4FE87]	1.6862	0.01921337
AAEL008031	AAEL008031	carbonic anhydrase	1.6844	0.00330646
AAEL004000	TOLL10	Toll-like receptor	1.6844	0.00046062
AAEL009127	CYP6M11	cytochrome P450	1.6843	6.37E-05
AAEL006064	AAEL006064	zinc finger protein	1.6827	0.01955115
AAEL003003	AAEL003003	glutamate-gated chloride channel	1.6818	2.01E-06
AAEL007129	AAEL007129	defective proboscis extension response, putative	1.6784	0.00149339
AAEL005714	AAEL005714	prolyl 4-hydroxylase alpha subunit 1,	1.6746	0.04931536
AAEL012383	AAEL012383	protein serine/threonine kinase, putative	1.6736	5.75E-05
AAEL009793	AAEL009793	cuticle protein, putative	1.6725	0.03103777
AAEL003183	AAEL003183	oxidoreductase	1.6722	0.00140149
AAEL007226	AAEL007226	nidogen	1.6721	0.02415986
AAEL001938	AAEL001938	ATP-binding cassette sub-family A member 3, putative	1.6671	0.03195419
AAEL000597	AAEL000597	cadherin	1.6642	3.84E-05
AAEL005052	AAEL005052	Tubulin beta chain [Source:UniProtKB/TrEMBL;Acc:Q17B46]	1.6635	0.02976933
AAEL007814	AAEL007814	n-twist	1.6634	0.00793434
AAEL013517	AAEL013517	pupal cuticle protein 78E, putative	1.6629	0.02811654
AAEL009191	AAEL009191	adenosine deaminase	1.6617	0.00029172

Gene ID	Gene Name	Description	log2FC	p-adj*
AAEL004935	AAEL004935	nicotinic acetylcholine receptor, beta-2 subunit, putative	1.6565	0.0504093
AAEL006267	AAEL006267	ras-related protein Rab-3C, putative	1.6552	0.00204521
AAEL005977	AAEL005977	chondroitin 4-sulfotransferase	1.6551	0.00098927
AAEL008069	AAEL008069	notch	1.6543	0.02743375
AAEL011368	AAEL011368	sugar transporter	1.6520	0.00214882
AAEL002417	AAEL002417	troponin t, invertebrate	1.6501	0.0011894
AAEL008354	AAEL008354	gaba receptor invertebrate	1.6490	6.51E-05
AAEL002761	AAEL002761	tropomyosin invertebrate	1.6456	0.00654924
AAEL010590	AAEL010590	aldose-1-epimerase	1.6413	0.03107126
AAEL004242	AAEL004242	juvenile hormone-inducible protein, putative	1.6406	0.00178887
AAEL011510	AAEL011510	multiple inositol polyphosphate phosphatase	1.6357	0.03321558
AAEL006167	AAEL006167	runt	1.6351	0.00223994
AAEL001457	AAEL001457	guanylatte cyclase alpha 1 subunit	1.6289	0.00049078
AAEL000910	AAEL000910	Fatty acyl-CoA reductase [Source:UniProtKB/TrEMBL;Acc:Q17MZ2]	1.6280	0.02362442
AAEL009266	AAEL009266	c4b-binding protein beta chain	1.6270	0.00048251
AAEL006572	AAEL006572	troponin C	1.6252	0.00167307
AAEL007104	AAEL007104	voltage-gated potassium channel	1.6211	9.55E-06
AAEL007764	AAEL007764	dipeptidyl-peptidase	1.6146	1.53E-05
AAEL006381	AAEL006381	sphingomyelin phosphodiesterase	1.6143	0.03791701
AAEL005207	AAEL005207	neprilysin	1.6106	5.49E-05
AAEL010381	AAEL010381	Glucosyl/glucuronosyl transferases [Source:UniProtKB/TrEMBL;Acc:A0A1S4FQK1]	1.6091	0.02981569
AAEL011716	AAEL011716	tartan	1.6057	0.00192808
AAEL010851	AAEL010851	Synaptotagmin-12 [Source:UniProtKB/TrEMBL;Acc:A0A1S4FS00]	1.6051	0.00018236

Gene ID	Gene Name	Description	log2FC	p-adj*
AAEL005619	AAEL005619	defective proboscis extension response, putative	1.6037	0.00015977
AAEL002572	AAEL002572	myosin regulatory light chain 2 (mlc-2)	1.6031	0.00300823
AAEL005964	AAEL005964	actin	1.6023	0.00017117
AAEL008658	LRIM16	leucine-rich immune protein (TM)	1.6010	0.01874286
AAEL001744	AAEL001744	jnk interacting protein (jip)	1.5983	0.00020095
AAEL007224	LRIM22	leucine-rich immune protein (Coil-less)	1.5977	0.00172163
AAEL014391	AAEL014391	Map kinase phosphatase [Source:UniProtKB/TrEMBL;Acc:A0A1S4G264]	1.5956	1.91E-05
AAEL007693	AAEL007693	organic anion transporter	1.5938	0.02512617
AAEL000244	AAEL000244	alpha(1,3)fucosyltransferase	1.5925	0.03031239
AAEL011784	AAEL011784	Alpha(1,3)fucosyltransferase [Source:UniProtKB/TrEMBL;Acc:A0A1S4FUB7]	1.5889	0.00270193
AAEL006276	AAEL006276	maltose phosphorylase	1.5862	0.03973941
AAEL011500	AAEL011500	Cdc42 protein, putative	1.5859	0.01607268
AAEL009024	GPRCAL3	GPCR Calcitonin/Diuretic Hormone Family	1.5840	0.00012287
AAEL000239	AAEL000239	clathrin coat assembly protein	1.5801	0.00050609
AAEL002005	CYP12F6	cytochrome P450	1.5794	0.0019481
AAEL013689	AAEL013689	neuronal calcium sensor, putative	1.5787	0.02947645
AAEL003977	AAEL003977	elongase, putative	1.5785	0.03026123
AAEL004295	AAEL004295	Glucose-6-phosphatase [Source:UniProtKB/TrEMBL;Acc:Q17D95]	1.5775	0.00684412
AAEL008032	AAEL008032	SET domain-containing protein [Source:UniProtKB/TrEMBL;Acc:A0A1S4FI78]	1.5745	2.50E-05
AAEL002583	TOLL7	Toll-like receptor	1.5697	0.00398572
AAEL002373	AAEL002373	juvenile hormone-inducible protein, putative	1.5681	0.04292839
AAEL002031	CYP12F7	cytochrome P450	1.5676	0.0106483
AAEL012873	AAEL012873	angiotensin-converting enzyme (dipeptidyl carboxypeptidase	1.5673	0.00073077

Gene ID	Gene Name	Description	log2FC	p-adj*
AAEL002231	AAEL002231	cuticle protein, putative	1.5645	0.04422898
AAEL003115	AAEL003115	tartan	1.5605	0.01394121
AAEL006815	CYP9J16	cytochrome P450	1.5601	0.03254821
AAEL014142	AAEL014142	phosphatidylinositol synthase	1.5591	0.0135993
AAEL000025	AAEL000025	phosphoenolpyruvate carboxykinase	1.5584	0.02149569
AAEL001816	AAEL001816	glucosyl/glucuronosyl transferases	1.5575	0.03928918
AAEL000092	GSTX1	glutathione transferase	1.5543	0.05019855
AAEL000405	AAEL000405	odd Oz protein	1.5534	0.00152673
AAEL002848	AAEL002848	tubulin beta chain	1.5524	4.00E-05
AAEL003914	AAEL003914	calcium/calmodulin-dependent serine protein kinase membrane-associated guanylate kinase (cask)	1.5507	0.00021846
AAEL004114	AAEL004114	UNC93A protein, putative	1.5484	0.04020998
AAEL005676	AAEL005676	adenosine deaminase	1.5445	0.01564184
AAEL008488	AAEL008488	cysteine-rich secretory protein-2, putative	1.5418	0.03489411
AAEL011727	AAEL011727	Molybdenum cofactor sulfurase 2 (MoCo sulfurase 2)(MOS 2)(EC 4.4.-.-)(Protein maroon-like 2)(Ma-l 2)	1.5398	0.04831722
AAEL011633	AAEL011633	fibrinogen and fibronectin	1.5395	0.00875869
AAEL007993	CLIPB27	Clip-Domain Serine Protease family B.	1.5359	0.04222212
AAEL012014	AAEL012014	L-lactate dehydrogenase [Source:UniProtKB/TrEMBL;Acc:Q16ND1]	1.5309	0.01507609
AAEL010739	AAEL010739	WNT4 precursor, putative	1.5281	0.01915905
AAEL009969	AAEL009969	axonemal dynein light chain	1.5254	0.0556839
AAEL017136	CYP325V1	cytochrome P450	1.5253	0.03978334
AAEL013251	AAEL013251	Protein quiver [Source:UniProtKB/TrEMBL;Acc:A0A1S4FYE1]	1.5095	0.01487604
AAEL003047	AAEL003047	netrin	1.5074	0.00256429
AAEL012911	LRIM18	leucine-rich immune protein (Coil-less)	1.5057	0.00126509

Gene ID	Gene Name	Description	log2FC	p-adj*
AAEL001912	AAEL001912	forkhead protein/ forkhead protein domain	1.5041	0.00838507
AAEL010676	AAEL010676	regulator of g protein signaling	1.5023	0.00500871
AAEL004416	AAEL004416	histone deacetylase	1.4983	0.01883703
AAEL003173	AAEL003173	forkhead protein/ forkhead protein domain	1.4968	0.03465909
AAEL010856	AAEL010856	dopamine beta hydroxylase	1.4954	0.00642127
AAEL010405	AAEL010405	alkyldihydroxyacetonephosphate synthase	1.4937	0.03004225
AAEL009244	AAEL009244	serine-type enodpeptidase,	1.4924	0.04497389
AAEL012056	ORP1	oxysterol-binding protein related protein (ORP1)	1.4897	0.00011054
AAEL000044	AAEL000044	ornithine decarboxylase	1.4867	0.0275475
AAEL009950	AAEL009950	homeotic deformed protein, putative	1.4863	0.03465909
AAEL011234	AAEL011234	reticulon/nogo receptor	1.4828	0.00659167
AAEL008055	AAEL008055	nicotinic acetylcholine receptor subunit	1.4794	0.00052923
AAEL012340	AAEL012340	lipase 1 precursor	1.4775	0.05331899
AAEL001568	AAEL001568	glycine receptor beta precursor, putative	1.4774	0.0259346
AAEL011193	AAEL011193	steroid dehydrogenase	1.4772	0.03545603
AAEL000948	AAEL000948	Tetraspanin [Source:UniProtKB/TrEMBL;Acc:A0A1S4EXC3]	1.4747	0.04468182
AAEL011056	AAEL011056	transcription factor HES-1 (Hairy and enhancer of split 1), putative	1.4728	0.0024407
AAEL011222	SCRB5	Class B Scavenger Receptor (CD36 domain).	1.4716	0.00822686
AAEL002575	ppk103	pickpocket 103	1.4715	0.03366731
AAEL011901	AAEL011901	1-acyl-sn-glycerol-3-phosphate acyltransferase	1.4705	0.05415785
AAEL002307	AAEL002307	leucine-rich transmembrane protein	1.4694	0.04692102
AAEL010793	AAEL010793	F-box/leucine rich repeat protein	1.4680	0.00302744

Gene ID	Gene Name	Description	log2FC	p-adj*
AAEL002321	AAEL002321	paired box protein pax-6	1.4678	0.00182393
AAEL000206	AAEL000206	ninjurin A, putative	1.4665	0.04631165
AAEL012575	AAEL012575	serine protease, putative	1.4658	0.04131842
AAEL005625	GPROP5	long wavelength sensitive opsin	1.4656	0.0478577
AAEL010479	AAEL010479	sugar transporter	1.4647	0.04503562
AAEL012110	AAEL012110	protease m1 zinc metalloprotease	1.4633	0.00012656
AAEL003194	AAEL003194	xaa-pro aminopeptidase	1.4611	0.00188956
AAEL014450	AAEL014450	Peptidyl-glycine alpha-amidating monooxygenase [Source:UniProtKB/TrEMBL;Acc:A0A1S4G1Z5]	1.4592	0.00136648
AAEL004616	AAEL004616	actin	1.4555	0.0571257
AAEL010142	AAEL010142	SBF2 domain-containing protein [Source:UniProtKB/TrEMBL;Acc:A0A1S4FPU2]	1.4523	0.00143626
AAEL011641	AAEL011641	transferrin	1.4517	0.04667575
AAEL003066	AAEL003066	brain chitinase and chia	1.4453	0.04219067
AAEL008817	AAEL008817	hexamerin 2 beta	1.4441	0.01262856
AAEL003301	AAEL003301	MRAS2, putative	1.4438	0.00525681
AAEL009760	AAEL009760	Niemann-Pick Type C-2, putative	1.4414	0.01115187
AAEL011078	CTLGA1	C-Type Lectin (CTL) - galactose binding.	1.4399	0.04450109
AAEL009045	AAEL009045	amine oxidase	1.4330	0.01463618
AAEL005604	AAEL005604	trypsin-epsilon, putative	1.4327	0.03056933
AAEL014125	AAEL014125	nk homeobox protein	1.4300	0.00058988
AAEL007024	CYP6AG3	cytochrome P450	1.4279	0.03998115
AAEL008109	AAEL008109	Protein mab-21-like	1.4276	0.01093367
AAEL015018	AAEL015018	toll	1.4244	0.05000647
AAEL001368	AAEL001368	kek1	1.4240	0.00193115
AAEL000715	AAEL000715	Zinc finger protein [Source:UniProtKB/TrEMBL;Acc:A0A1S4EWM9]	1.4237	0.00841728

Gene ID	Gene Name	Description	log2FC	p-adj*
AAEL011905	AAEL011905	myosin i	1.4219	0.04484124
AAEL014605	CYP9J9	cytochrome P450	1.4208	0.01150815
AAEL011741	GSTS1	glutathione transferase	1.4206	0.06360717
AAEL000687	AAEL000687	glucosyl/glucuronosyl transferases	1.4201	0.03484901
AAEL012069	GPXH1	glutathione peroxidase	1.4177	0.03998115
AAEL010041	AAEL010041	SH2 domain-containing protein [Source:UniProtKB/TrEMBL;Acc:A0A1S4FPI0]	1.4146	0.00922033
AAEL008046	AAEL008046	rh antigen	1.4144	0.01683543
AAEL006070	AAEL006070	Phosphoglycerate mutase [Source:UniProtKB/TrEMBL;Acc:Q177P3]	1.4143	0.00058339
AAEL012128	AAEL012128	cationic amino acid transporter	1.4130	0.00815629
AAEL012326	AAEL012326	calmodulin	1.4125	0.01877416
AAEL006902	AAEL006902	serine-type enodpeptidase,	1.4083	0.03117753
AAEL004509	AAEL004509	Kynurenine formamidase [Source:UniProtKB/TrEMBL;Acc:A0A1S4F7T3]	1.4074	0.04157526
AAEL001864	AAEL001864	eukaryotic translation initiation factor 4E binding protein (4EBP)	1.4074	0.00191883
AAEL000314	AAEL000314	malate dehydrogenase	1.4059	0.02272957
AAEL006980	AAEL006980	lipase	1.4042	0.02947645
AAEL010224	AAEL010224	transcription factor GATA-4 (GATA binding factor-4)	1.4029	0.00571519
AAEL014700	AAEL014700	sugar transporter	1.4001	0.04896874
AAEL011522	AAEL011522	MRAS2, putative	1.3997	0.00183587
AAEL004575	AAEL004575	beta-galactosidase	1.3992	0.00391329
AAEL009777	AAEL009777	heparan sulphate sulfotransferase	1.3977	0.00964818
AAEL003910	AAEL003910	adam	1.3946	0.01380448
AAEL006984	CYP6AG5	cytochrome P450	1.3941	0.04292839
AAEL005187	AAEL005187	laminin gamma 1 chain	1.3930	0.0184361
AAEL000340	AAEL000340	cytochrome P450	1.3883	0.05489642

Gene ID	Gene Name	Description	log2FC	p-adj*
AAEL009117	CYP6M5	cytochrome P450	1.3859	0.0307846
AAEL008056	AAEL008056	hyperpolarization activated cyclic nucleotide-gated potassium channel	1.3845	0.00311979
AAEL010850	AAEL010850	troponin i	1.3845	0.00203624
AAEL002678	AAEL002678	ras suppressor protein 1, rsu1	1.3843	0.01144696
AAEL003408	AAEL003408	leucine-rich transmembrane protein	1.3834	0.02947645
AAEL008117	AAEL008117	ion channel nompc	1.3804	0.05674222
AAEL001127	AAEL001127	defective proboscis extension response, putative	1.3796	0.00328744
AAEL008628	AAEL008628	ABC transporter	1.3769	0.02764848
AAEL017498	CASPS21	caspase	1.3755	0.03252845
AAEL009601	AAEL009601	pyridoxine kinase	1.3751	0.05746656
AAEL009858	SIFa	SIFamide	1.3736	0.00571519
AAEL006014	HPX1	heme peroxidase	1.3734	0.05806602
AAEL000298	AAEL000298	sodium/chloride dependent amino acid transporter	1.3719	0.00011666
AAEL006490	GPRCAL2	GPCR Calcitonin/Diuretic Hormone Family	1.3715	0.01069627
AAEL009421	AAEL009421	cyclophilin-r	1.3710	0.05016851
AAEL006254	GlcAT-P	beta-1,3-glucuronyltransferase s, p	1.3701	7.97E-05
AAEL007913	AAEL007913	sulfotransferase (sult)	1.3689	0.0629718
AAEL006439	AAEL006439	testis/ seletal muscle dual specificty phosphatase	1.3675	0.00074124
AAEL008342	AAEL008342	monocarboxylate transporter	1.3652	0.03884132
AAEL012139	AAEL012139	allatostatin, B-type, putative	1.3649	0.00196383
AAEL009847	AAEL009847	microtubule-associated protein	1.3614	0.00029063
AAEL006160	AAEL006160	runx1 (aml1)	1.3611	0.00049817
AAEL003967	AAEL003967	calpain 4, 6, 7, invertebrate	1.3558	0.04552812
AAEL003129	AAEL003129	neuroligin,	1.3552	0.0259346

Gene ID	Gene Name	Description	log2FC	p-adj*
AAEL008271	AAEL008271	SEC14, putative	1.3540	0.04047256
AAEL002571	AAEL002571	band 4.1-like protein 4A (NBL4 protein), putative	1.3539	0.0009849
AAEL010772	AAEL010772	Leucine-rich repeat-containing protein 50 homolog	1.3533	0.06292019
AAEL006805	CYP9J2	cytochrome P450	1.3515	0.00136546
AAEL010963	AAEL010963	brain chitinase and chia	1.3495	0.07064081
AAEL003444	CASPS19	caspase (short)	1.3469	0.04484124
AAEL012135	GALE2	Galectin [Source:UniProtKB/TrEMBL;Acc:Q16MZ7]	1.3462	0.05547256
AAEL003713	AAEL003713	leucine-rich transmembrane protein	1.3455	0.00246671
AAEL015404	LYSC7B	C-Type Lysozyme (Lys-B).	1.3450	0.0361525
AAEL008699	AAEL008699	prolylcarboxypeptidase, putative	1.3449	0.05878157
AAEL007029	AAEL007029	tropomodulin	1.3443	0.04559898
AAEL012384	AAEL012384	amalgam protein, putative	1.3407	0.0064056
AAEL004025	AAEL004025	glucose dehydrogenase	1.3406	0.05253516
AAEL001373	AAEL001373	lim homeobox protein	1.3405	0.00214882
AAEL001091	AAEL001091	Malic enzyme [Source:UniProtKB/TrEMBL;Acc:A0A1S4EXR8]	1.3398	0.01363114
AAEL004174	AAEL004174	T-box transcription factor tbx6	1.3388	0.00748652
AAEL002343	AAEL002343	neuronal pas domain protein	1.3359	0.00890194
AAEL003026	AAEL003026	regulator of g protein signaling	1.3349	0.00013653
AAEL012072	AAEL012072	Histone H3 [Source:UniProtKB/TrEMBL;Acc:Q16N56]	1.3330	0.08010791
AAEL001586	AAEL001586	glucosyl/glucuronosyl transferases	1.3316	0.03346418
AAEL006995	AAEL006995	sodium/solute symporter	1.3274	0.00763566
AAEL001920	AAEL001920	SET domain-containing protein [Source:UniProtKB/TrEMBL;Acc:A0A1S4F084]	1.3211	0.04635023
AAEL013983	AAEL013983	hexamerin 2 beta	1.3206	0.06870234
AAEL017301	AAEL017301	Elongation factor 1-alpha [Source:UniProtKB/TrEMBL;Acc:J9HYQ9]	1.3179	0.0134256

Gene ID	Gene Name	Description	log2FC	p-adj*
AAEL003966	AAEL003966	lachesin, putative	1.3176	0.01754102
AAEL002196	AAEL002196	Procathepsin L3, putative [Source:UniProtKB/TrEMBL;Acc:A0A1S4F176]	1.3161	0.01761467
AAEL000820	AAEL000820	dimethylaniline monooxygenase	1.3137	0.03733289
AAEL001232	AAEL001232	tubulointerstitial nephritis antigen	1.3115	1.63E-06
AAEL010348	AAEL010348	sugar transporter	1.3110	0.0605646
AAEL004457	AAEL004457	cytochrome c	1.3106	0.04359101
AAEL013648	AAEL013648	Fatty acyl-CoA reductase [Source:UniProtKB/TrEMBL;Acc:Q16II8]	1.3104	0.01390933
AAEL008303	AAEL008303	calponin/transgelin	1.3102	0.01884834
AAEL002658	AAEL002658	AMP dependent ligase	1.3100	0.00969517
AAEL000714	AAEL000714	sodium-dependent excitatory amino acid transporter	1.3086	0.00172913
AAEL015283	AAEL015283	synaptic vesicle protein	1.2977	0.06142111
AAEL002741	SCRB6	Class B Scavenger Receptor (CD36 domain).	1.2974	0.05858623
AAEL001695	AAEL001695	orthopedia homeobox protein	1.2929	0.00262134
AAEL011643	AAEL011643	homothorax homeobox protein	1.2928	0.00035717
AAEL007785	AAEL007785	leucine-rich transmembrane protein	1.2915	0.01427357
AAEL007146	AAEL007146	voltage-gated potassium channel	1.2889	0.0053334
AAEL011979	AAEL011979	calmodulin	1.2882	0.04618429
AAEL008278	AAEL008278	CRAL-TRIO domain-containing protein [Source:UniProtKB/TrEMBL;Acc:A0A1S4FJA2]	1.2840	0.03822893
AAEL010975	AAEL010975	paramyosin, long form	1.2838	0.00896751
AAEL003387	AAEL003387	cationic amino acid transporter	1.2836	0.01797585
AAEL001167	AAEL001167	synaptotagmin	1.2829	0.00080977
AAEL014137	CLIPB25	Clip-Domain Serine Protease family B.	1.2823	0.00192421
AAEL000642	AAEL000642	alpha-amylase	1.2816	0.00803013

Gene ID	Gene Name	Description	log2FC	p-adj*
AAEL011206	AAEL011206	aminoacylase, putative	1.2794	0.04784205
AAEL005992	AAEL005992	adam (a disintegrin and metalloprotease)	1.2790	0.02431284
AAEL009588	AAEL009588	expressed protein (HR3)	1.2787	0.00401235
AAEL005109	AAEL005109	WD-repeat protein	1.2780	0.07188577
AAEL003837	AAEL003837	ryanodine receptor 3, brain	1.2771	0.00195158
AAEL013505	GPRNPY7	GPCR Neuropeptide Y Family	1.2771	0.00134896
AAEL011638	AAEL011638	Cyclic-nucleotide-gated cation channel [Source:UniProtKB/TrEMBL;Acc:A0A1S4FU57]	1.2761	0.00227911
AAEL006648	AAEL006648	pantothenate kinase	1.2737	0.03421494
AAEL014255	AAEL014255	aquaporin, putative	1.2724	0.00088065
AAEL004947	AAEL004947	elongase, putative	1.2710	0.07278413
AAEL008202	AAEL008202	serine-type enodpeptidase,	1.2708	0.07239784
AAEL002673	AAEL002673	elongase, putative	1.2689	0.0002014
AAEL006615	AAEL006615	zinc finger protein	1.2685	0.00642127
AAEL014840	AAEL014840	short-chain dehydrogenase	1.2672	0.07277722
AAEL000726	AAEL000726	fibrinogen and fibronectin	1.2671	0.01619106
AAEL002614	AAEL002614	equilibrative nucleoside transporter	1.2671	0.01920493
AAEL005177	AAEL005177	adenylate cyclase	1.2616	0.03876269
AAEL004602	AAEL004602	neurogenic differentiation factor, putative	1.2609	0.05193279
AAEL002606	OBP35	odorant binding protein OBP35	1.2599	0.00534784
AAEL012693	AAEL012693	tripartite motif protein	1.2547	0.07076025
AAEL008654	AAEL008654	ZP domain-containing protein [Source:UniProtKB/TrEMBL;Acc:A0A1S4FK14]	1.2535	0.07431208
AAEL005536	AAEL005536	tetraspanin 29fb	1.2532	0.04784205
AAEL006287	PIWI7	PIWI	1.2497	0.04985249
AAEL010868	AAEL010868	sugar transporter	1.2493	0.04112181

Gene ID	Gene Name	Description	log2FC	p-adj*
AAEL007205	AAEL007205	gata-binding factor-c (transcription factor gata-c)	1.2485	0.03977052
AAEL008014	AAEL008014	ef hand protein	1.2482	0.00702556
AAEL004825	AAEL004825	potassium-dependent sodium-calcium exchanger, putative	1.2480	0.0113771
AAEL012207	AAEL012207	myosin light chain 1,	1.2478	0.00183587
AAEL013063	APG18A	autophagy related gene	1.2466	0.01326033
AAEL017071	AAEL017071	Carboxylic ester hydrolase [Source:UniProtKB/TrEMBL;Acc:A0A1S4G5Q9]	1.2461	0.0749261
AAEL009240	AAEL009240	neurotactin	1.2455	0.00255465
AAEL000188	AAEL000188	elongase, putative	1.2451	0.00143414
AAEL001813	AAEL001813	sodium/solute symporter	1.2450	0.03600672
AAEL002141	AAEL002141	endothelin-converting enzyme	1.2399	0.00280296
AAEL000372	AAEL000372	myo inositol monophosphatase	1.2385	0.05699017
AAEL010172	AAEL010172	Leucokinins Precursor [Contains Leucokinin-1;Leucokinin-3;Leucokinin-2]	1.2372	0.00718906
AAEL010105	AAEL010105	MRAS2, putative	1.2367	0.01340384
AAEL012712	CLIPC13	Clip-Domain Serine Protease family C.	1.2366	0.00707498
AAEL006478	AAEL006478	lachesin, putative	1.2353	0.00456531
AAEL008765	AAEL008765	cuticle protein, putative	1.2350	0.07713222
AAEL009055	AAEL009055	gliotactin	1.2344	0.03163153
AAEL012120	AAEL012120	fad oxidoreductase	1.2343	0.0339507
AAEL008847	AAEL008847	wingless	1.2342	0.0006332
AAEL013197	AAEL013197	Chitin-binding type-4 domain-containing protein [Source:UniProtKB/TrEMBL;Acc:A0A1S4FYP2]	1.2324	0.0565072
AAEL007719	AAEL007719	rhombotin	1.2323	0.00068402
AAEL003111	AAEL003111	tartan	1.2319	0.01855285
AAEL009464	AAEL009464	knolle, putative	1.2304	0.05598484

Gene ID	Gene Name	Description	log2FC	p-adj*
AAEL013798	CYP4H33	cytochrome P450	1.2297	0.0513197
AAEL012621	AAEL012621	arginine/serine-rich splicing factor	1.2257	0.04744636
AAEL012740	AAEL012740	ATPase subunit, putative	1.2207	0.01042103
AAEL013281	AAEL013281	serine-type enodpeptidase	1.2183	0.06307895
AAEL017549	GPRNNA14	GPCR Orphan/Putative Class A Family	1.2173	0.03624346
AAEL005040	AAEL005040	E3 ubiquitin-protein ligase [Source:UniProtKB/TrEMBL;Acc:Q17BB8]	1.2169	0.06047951
AAEL019691	sNPF	Short neuropeptide F [Source:UniProtKB/Swiss-Prot;Acc:A0SIX6]	1.2115	0.00940254
AAEL000533	CTL16	C-Type Lectin (CTL16)	1.2112	0.05601897
AAEL000704	AAEL000704	synaptotagmin,	1.2112	0.00202489
AAEL002538	AAEL002538	glutamate receptor, ionotropic kainate 1, 2, 3 (glur5, glur6, glur7)	1.2097	0.00370641
AAEL009295	AAEL009295	lachesin	1.2088	0.00215494
AAEL013405	AAEL013405	nk homeobox protein	1.2082	0.05152906
AAEL006829	AAEL006829	microsomal glutathione s-transferase	1.2067	0.05752618
AAEL006214	AAEL006214	PIP49_N domain-containing protein [Source:UniProtKB/TrEMBL;Acc:A0A1S4FCV1]	1.2066	0.01676791
AAEL011088	Dat	dopamine N-acetyltransferase	1.2059	0.06047951
AAEL027982	PGRP-LE	peptidoglycan recognition protein LE	1.2029	0.00713828
AAEL001022	AAEL001022	anterior fat body protein	1.2022	0.07884715
AAEL008857	AAEL008857	deoxyribonuclease I, putative	1.2020	0.07213935
AAEL001628	AAEL001628	DUF4808 domain-containing protein [Source:UniProtKB/TrEMBL;Acc:A0A1S4EZB6]	1.2011	0.00121658
AAEL012783	AAEL012783	protease m1 zinc metalloprotease	1.2011	0.05699017
AAEL012298	AAEL012298	sterol desaturase	1.2001	0.00933449
AAEL004098	AAEL004098	neuromusculin	1.1989	0.00381309
AAEL002360	AAEL002360	serine-type enodpeptidase,	1.1967	0.07034406
AAEL001673	AAEL001673	actin	1.1935	0.0372937
AAEL004119	AAEL004119	ribonuclease p/mrp subunit	1.1919	0.00282901

Gene ID	Gene Name	Description	log2FC	p-adj*
AAEL006476	AAEL006476	symbol, putative	1.1918	0.02858129
AAEL010384	AAEL010384	aldehyde oxidase	1.1912	0.08128063
AAEL012450	AAEL012450	serine/threonine-protein kinase	1.1849	0.00029125
AAEL012114	AAEL012114	troponin C	1.1843	0.03059421
AAEL003104	AAEL003104	tripartite motif protein trim2,3	1.1829	0.02473704
AAEL001900	AAEL001900	lactosylceramide 4-alpha-galactosyltransferase (alpha- 1,4-galactosyltransferase)	1.1814	0.06304718
AAEL011112	AAEL011112	alcohol dehydrogenase	1.1769	0.01435143
AAEL005006	CYP6CD1	cytochrome P450	1.1767	0.08326663
AAEL000652	GNBPA2	Gram-Negative Binding Protein (GNBP) or Beta-1 3-Glucan Binding Protein (BGBP).	1.1754	0.06694282
AAEL002273	AAEL002273	trypsin, putative	1.1722	0.0835038
AAEL011280	AAEL011280	voltage-dependent p/q type calcium channel	1.1719	0.00676112
AAEL008609	AAEL008609	zinc carboxypeptidase	1.1718	0.05634841
AAEL005014	AAEL005014	transient receptor potential channel	1.1693	0.01134845
AAEL009792	LRIM25	leucine-rich immune protein (Coil-less)	1.1691	0.05842933
AAEL006718	AAEL006718	Sugar transporter SWEET [Source:UniProtKB/TrEMBL;Acc:Q174X0]	1.1679	0.07243178
AAEL005793	AAEL005793	AMP dependent ligase	1.1673	0.00074124
AAEL012522	AAEL012522	Sodium-dependent phosphate transporter [Source:UniProtKB/TrEMBL;Acc:A0A1S4FWV3]	1.1668	0.06903756
AAEL004299	AAEL004299	angiotensin-converting-relatedenzyme	1.1666	0.06777641
AAEL002626	AAEL002626	odorant-binding protein 56e, putative	1.1665	0.06905363
AAEL003891	AAEL003891	Ctl transporter [Source:UniProtKB/TrEMBL;Acc:A0A1S4F6B7]	1.1651	0.04767149
AAEL019881	AAEL019881	Sex peptide receptor [Source:UniProtKB/TrEMBL;Acc:B0F4E9]	1.1638	0.00384475
AAEL005345	AAEL005345	transcription factor castor (protein ming)	1.1625	0.0229865

Gene ID	Gene Name	Description	log2FC	p-adj*
AAEL010853	AAEL010853	AMP dependent ligase	1.1576	0.05802279
AAEL000234	SCRB7	Class B Scavenger Receptor (CD36 domain).	1.1540	0.06939831
AAEL013441	TOLL9A	Toll-like receptor	1.1524	0.06000219
AAEL007055	AAEL007055	lipase	1.1515	0.07563597
AAEL011007	AAEL011007	fibrinogen and fibronectin	1.1508	0.00126509
AAEL005979	SCRB3	Class B Scavenger Receptor (CD36 domain).	1.1497	0.04290839
AAEL002055	AAEL002055	neuroendocrine protein 7b2	1.1493	0.01012152
AAEL001209	AAEL001209	sodium-dependent phosphate transporter	1.1485	0.07914413
AAEL008190	AAEL008190	bhlh factor math6 (atoh8 protein) (bhlh transcription factor)	1.1463	0.01053152
AAEL003423	AAEL003423	NADH dehydrogenase, putative	1.1446	0.00106531
AAEL004876	AAEL004876	guanylate cyclase	1.1444	0.04417825
AAEL010145	AAEL010145	sodium/potassium-dependent ATPase beta-2 subunit	1.1438	0.08082691
AAEL000265	AAEL000265	Protein crossbronx homolog	1.1413	0.0259346
AAEL011915	AAEL011915	F-box/lrr protein fragment	1.1413	0.04397564
AAEL008840	AAEL008840	Bardet-Biedl syndrome 2 protein homolog [Source:UniProtKB/TrEMBL;Acc:Q16XJ9]	1.1406	0.06044004
AAEL007299	AAEL007299	Cadherin, putative [Source:UniProtKB/TrEMBL;Acc:A0A1S4FG21]	1.1400	0.03252845
AAEL013407	Cat	Catalase [Source:UniProtKB/TrEMBL;Acc:Q16J86]	1.1384	0.07507897
AAEL014618	AAEL014618	cytochrome P450	1.1363	0.03405388
AAEL006947	GPRTAK2	GPCR Neurokinin/Tachykinin Family	1.1351	0.00722505
AAEL009298	AAEL009298	2-Keto-3-deoxy-(6-phospho-)gluconate aldolase, putative	1.1344	0.04069733
AAEL012886	AAEL012886	Carboxylic ester hydrolase (Fragment) [Source:UniProtKB/TrEMBL;Acc:Q16KS8]	1.1326	0.00765408
AAEL003154	AAEL003154	aldo-keto reductase	1.1321	0.06529728

Gene ID	Gene Name	Description	log2FC	p-adj*
AAEL004313	AAEL004313	fk506-binding protein	1.1289	0.03025306
AAEL001374	AAEL001374	flagellar radial spoke protein	1.1269	0.01515248
AAEL003831	AAEL003831	fatty acid hydroxylase	1.1266	0.08599036
AAEL005957	AAEL005957	phospholipase b, plb1	1.1264	0.08300243
AAEL012060	AAEL012060	PBAN-type neuropeptides Precursor (Pheromone/pyrokinin biosynthesis-activating neuropeptide)	1.1258	0.01469425
AAEL002555	AAEL002555	sodium/solute symporter	1.1244	0.0891674
AAEL008588	inx7	Innexin [Source:UniProtKB/TrEMBL;Acc:Q16YE3]	1.1225	0.09101556
AAEL010354	AAEL010354	homeobox protein nk-2	1.1222	0.0901422
AAEL016984	AAEL016984	Glyceraldehyde-3-phosphate dehydrogenase [Source:UniProtKB/TrEMBL;Acc:J9HYM2]	1.1217	0.03737249
AAEL005043	AAEL005043	ATP-dependent bile acid permease	1.1205	0.02079285
AAEL008421	AAEL008421	cadherin	1.1205	0.04510651
AAEL009183	AAEL009183	cytochrome oxidase biogenesis protein (oxa1 mitochondrial)	1.1202	0.06401658
AAEL009183	AAEL009183	cytochrome oxidase biogenesis protein (oxa1 mitochondrial)	1.1202	0.06401658
AAEL002397	AAEL002397	DUF2236 domain-containing protein [Source:UniProtKB/TrEMBL;Acc:A0A1S4F1N1]	1.1197	0.07026435
AAEL004931	AAEL004931	beta-hexosaminidase b	1.1180	0.03227015
AAEL005195	AAEL005195	homeobox protein	1.1160	0.07625905
AAEL004646	AAEL004646	actin	1.1151	0.08583753
AAEL009949	AAEL009949	homeotic antennapedia protein, putative	1.1142	0.01278897
AAEL004631	AAEL004631	actin	1.1120	0.03465068
AAEL003234	AAEL003234	cytochrome c oxidase, subunit VIA, putative	1.1081	0.00517525
AAEL010043	GPRCAL1	GPCR Calcitonin/Diuretic Hormone Family	1.1078	0.03046289

Gene ID	Gene Name	Description	log2FC	p-adj*
AAEL002754	AAEL002754	nephrin	1.1076	0.01710716
AAEL001844	AAEL001844	zinc carboxypeptidase	1.1071	0.08034457
AAEL006171	AAEL006171	n-myc downstream regulated	1.1062	0.02579543
AAEL012164	SPZ6	spaetzle-like cytokine	1.1054	0.08329506
AAEL000164	AAEL000164	d-amino acid oxidase	1.1052	0.09347631
AAEL009991	AAEL009991	myosin iii	1.1032	0.00021284
AAEL006482	AAEL006482	sugar transporter	1.1009	0.01483677
AAEL007221	AAEL007221	Brain-specific homeobox protein, putative [Source:UniProtKB/TrEMBL;Acc:A0A1S4FG44]	1.1003	0.03465909
AAEL008452	AAEL008452	alpha-amylase	1.1002	0.06119191
AAEL020382	AAEL020382	Acetyl-coenzyme A synthetase [Source:UniProtKB/TrEMBL;Acc:Q16EU8]	1.0996	0.00720497
AAEL007018	AAEL007018	udp-glucose 4-epimerase	1.0991	0.06864132
AAEL001914	AAEL001914	scavenger receptor, putative	1.0967	0.03872239
AAEL003645	AAEL003645	stearoyl-coa desaturase	1.0955	0.07721768
AAEL010823	AAEL010823	ATP synthase delta chain	1.0924	0.00725813
AAEL014965	AAEL014965	nova	1.0917	0.08471994
AAEL008887	AAEL008887	cytochrome c oxidase,-subunit VIb	1.0889	0.03258698
AAEL000516	AAEL000516	juvenile hormone-inducible protein, putative	1.0881	0.07213935
AAEL006095	AAEL006095	Gelsolin precursor	1.0878	0.09614662
AAEL012126	AAEL012126	F-box/lrr protein, drome	1.0811	0.0047203
AAEL002887	arm	Armadillo segment polarity protein [Source:UniProtKB/Swiss-Prot;Acc:Q17GS9]	1.0788	0.03791701
AAEL010408	GPRGBB2	GPCR GABA B Family	1.0737	0.03054403
AAEL006342	AAEL006342	epoxide hydrolase	1.0733	0.05466979
AAEL003214	AAEL003214	salivary gland growth factor	1.0725	0.09162713
AAEL000138	AAEL000138	NADH dehydrogenase, putative	1.0711	0.00209543

Gene ID	Gene Name	Description	log2FC	p-adj*
AAEL003809	AAEL003809	sugar transporter	1.0709	0.09916801
AAEL011871	AAEL011871	cytochrome C1	1.0680	0.02431284
AAEL001963	AAEL001963	protein serine/threonine kinase, putative	1.0671	0.05234859
AAEL004165	AAEL004165	bone morphogenetic protein receptor type II, putative	1.0663	0.00034934
AAEL011436	AAEL011436	myosin xv	1.0647	0.02974024
AAEL008624	AAEL008624	ABC transporter	1.0637	0.09790408
AAEL012549	AAEL012549	ATPase, class vi, type 11c	1.0626	0.09671571
AAEL006359	AAEL006359	sulfotransferase (sult)	1.0622	0.0850897
AAEL007548	AAEL007548	leukocyte receptor cluster (lrc) member	1.0612	0.04744636
AAEL002367	CCEAE1B	Carboxy/choline esterase Alpha Esterase	1.0548	0.05781392
AAEL005460	AAEL005460	alpha-galactosidase/alpha-n-acetylgalactosaminidase	1.0523	0.07293429
AAEL002745	AAEL002745	nucleoprotein, putative	1.0521	0.0457239
AAEL011954	AAEL011954	elongase, putative	1.0515	0.0845891
AAEL001433	AAEL001433	fgf receptor activating protein	1.0513	0.00230794
AAEL008062	AAEL008062	thrombospondin	1.0512	0.0779711
AAEL007054	AAEL007054	NADH dehydrogenase, putative	1.0498	0.00539213
AAEL014604	AAEL014604	cytochrome P450	1.0478	0.08393343
AAEL005666	AAEL005666	matrix metalloproteinase	1.0467	0.07229287
AAEL004357	AAEL004357	neuroligin,	1.0465	0.02362442
AAEL014847	inx2	Innexin [Source:UniProtKB/TrEMBL;Acc:Q16FA2]	1.0453	0.07288442
AAEL013640	AAEL013640	lung carbonyl reductase	1.0450	0.07043272
AAEL006948	AAEL006948	tomosyn	1.0440	0.01863844
AAEL009458	AAEL009458	syntaxin 4	1.0421	0.09823103
AAEL007293	AAEL007293	cAMP-dependent protein kinase catalytic subunit	1.0418	0.07398033

Gene ID	Gene Name	Description	log2FC	p-adj*
AAEL004229	GSTT4	glutathione transferase	1.0392	0.05878157
AAEL004507	AAEL004507	valacyclovir hydrolase	1.0384	0.04641279
AAEL003560	AAEL003560	pou domain/drifter/cf-1a	1.0378	0.0217352
AAEL006509	AAEL006509	ATP synthase subunit [Source:UniProtKB/TrEMBL;Acc:Q1HR38]	1.0376	0.0103773
AAEL002335	AAEL002335	atbf1	1.0358	0.046831
AAEL011221	AAEL011221	nmda receptor glutamate-binding chain	1.0354	0.07507897
AAEL007868	AAEL007868	ubiquinol-cytochrome c reductase complex 14 kd protein	1.0335	0.02089214
AAEL011488	AAEL011488	short-chain dehydrogenase	1.0329	0.08776928
AAEL010941	AAEL010941	Protein tweety homolog [Source:UniProtKB/TrEMBL;Acc:Q16RK1]	1.0329	0.00479242
AAEL008827	AAEL008827	cell adhesion molecule	1.0319	0.00015617
AAEL006232	GPRNND3	GPCR Orphan/Putative Class D Family	1.0317	0.00300188
AAEL009066	AAEL009066	symbol, putative	1.0312	0.02268198
AAEL009173	AAEL009173	fasciclin ii (fas ii)	1.0310	0.04292839
AAEL018207	CTL8	C-Type lectin (CTL)	1.0287	0.04163423
AAEL003873	AAEL003873	Glycerol-3-phosphate dehydrogenase [Source:UniProtKB/TrEMBL;Acc:Q17E82]	1.0273	0.03749144
AAEL007794	AAEL007794	defective proboscis extension response	1.0269	0.03861071
AAEL011763	PPO3	prophenoloxidase	1.0266	0.07484293
AAEL007770	AAEL007770	voltage and ligand gated potassium channel	1.0249	0.05467909
AAEL002456	AAEL002456	b-cell lymphoma/leukemia	1.0236	0.00602353
AAEL009682	AAEL009682	serine collagenase 1 precursor, putative	1.0227	0.09638847
AAEL008871	AAEL008871	succinate dehydrogenase	1.0225	0.03570141
AAEL003139	AAEL003139	short-chain dehydrogenase	1.0211	0.08480194
AAEL014589	AAEL014589	reticulocalbin	1.0130	0.06968499
AAEL001210	AAEL001210	NADH ubiquinone oxidoreductase subunit, putative	1.0098	0.00215432

Gene ID	Gene Name	Description	log2FC	p-adj*
AAEL011992	AAEL011992	NADH:ubiquinone dehydrogenase, putative	1.0082	0.00716938
AAEL004513	AAEL004513	neurotransmitter gated ion channel	1.0074	0.08128063
AAEL000523	AAEL000523	ring finger protein	1.0049	0.04767149
AAEL007940	Gr77	gustatory receptor Gr77	1.0042	0.07933505
AAEL014138	SRPN16	Serine Protease Inhibitor (serpin) likely cleavage at S/L.	1.0033	0.06929267
AAEL009126	CYP6N6	cytochrome P450	1.0021	0.08542362
AAEL009131	CYP6Z8	cytochrome P450	1.0001	0.09671571

*adjusted by Benjamini-Hochberg correction method

Table S5. List of significantly downregulated annotated genes in field-G0 samples relative to UPLB-UNXP-G122 samples from DESeq2 v.1.46.0 in R Studio v.2024.12.0+467

Gene ID	Gene Name	Description	log2FC	p-adj*
AAEL018669	COX3	cytochrome c oxidase subunit III	-7.9379	1.2E-13
AAEL018681	ND4L	NADH dehydrogenase subunit 4L	-6.3261	0.0001
AAEL013422	AAEL013422	odorant receptor 42a, putative	-4.5737	1.2E-06
AAEL018658	ND2	NADH dehydrogenase subunit 2	-4.0615	0.0075
AAEL004975	AAEL004975	Zinc finger protein [Source:UniProtKB/TrEMBL;Acc:A0A1S4F966]	-3.6295	0.0018
AAEL026460	AAEL026460	Histone H3 [Source:UniProtKB/TrEMBL;Acc:Q16N56]	-3.2847	0.0053
AAEL012565	AAEL012565	lumbrokinase-3(1) precursor, putative	-3.0143	0.0371
AAEL014544	PPO6	prophenoloxidase	-3.0036	1.0E-05
AAEL015682	AAEL015682	Histone H2B [Source:UniProtKB/TrEMBL;Acc:Q17ER2]	-2.9715	0.0029
AAEL004028	AAEL004028	glucose dehydrogenase	-2.7848	0.0026
AAEL009149	AAEL009149	kinectin, putative	-2.6883	1.8E-05
AAEL005691	AAEL005691	protein serine/threonine kinase, putative	-2.6498	0.0079

Gene ID	Gene Name	Description	log2FC	p-adj*
AAEL013198	AAEL013198	ionotropic glutamate receptor-invertebrate	-2.5987	0.0214
AAEL009122	AAEL009122	cytochrome P450	-2.5583	0.0055
AAEL007097	AAEL007097	4-nitrophenylphosphatase [Source:UniProtKB/TrEMBL;Acc:Q0IF18]	-2.5155	0.0068
AAEL019789	AAEL019789	40S ribosomal protein S3a [Source:UniProtKB/Swiss-Prot;Acc:Q1HRR3]	-2.3264	9.7E-09
AAEL002639	Rpl36-2	60S ribosomal protein L36 [Source:UniProtKB/TrEMBL;Acc:Q17HK5]	-2.2389	0.0002
AAEL003669	AAEL003669	Histone H2A [Source:UniProtKB/TrEMBL;Acc:Q17ER3]	-2.1722	5.8E-05
AAEL007013	AAEL007013	RNA-binding protein precursor, putative	-2.1061	0.0034
AAEL009749	AAEL009749	Sodium-dependent phosphate transporter [Source:UniProtKB/TrEMBL;Acc:A0A1S4FN96]	-2.0486	0.0067
AAEL003289	AAEL003289	alkaline phosphatase	-1.9061	0.04809177
AAEL015458	Tf1	transferrin	-1.8861	0.02994817
AAEL008427	AAEL008427	smad nuclear interacting protein	-1.8658	0.000102
AAEL000563	mosGCTL-1	C-Type Lectin (CTL) - mannose binding.	-1.8223	0.01610975
AAEL013912	AAEL013912	Fatty acyl-CoA reductase [Source:UniProtKB/TrEMBL;Acc:A0A1S4G0R7]	-1.7696	0.01017743
AAEL004245	GPRNNA19_1	GPCR Orphan/Putative Class A Family	-1.7391	0.06636256
AAEL004104	AAEL004104	F-box domain-containing protein [Source:UniProtKB/TrEMBL;Acc:A0A1S4F6M5]	-1.7021	0.05155219
AAEL003631	CLIPB41	Clip-Domain Serine Protease family B.	-1.6974	0.00249039
AAEL004036	AAEL004036	glucose dehydrogenase	-1.6806	0.00465566
AAEL004402	AAEL004402	alpha-l-iduronidase	-1.6181	3.10E-05
AAEL006282	AAEL006282	tetraspanin, putative	-1.5983	0.00454843
AAEL012431	AAEL012431	AMP dependent ligase	-1.5860	0.00803013
AAEL017005	GPRNPY6	GPCR Neuropeptide Y Family	-1.5679	0.03545603

Gene ID	Gene Name	Description	log2FC	p-adj*
AAEL028129	AAEL028129	Putative nuclear transcription factor y subunit gamma [Source:UniProtKB/TrEMBL;Acc:A0A0P6K0W4]	-1.5609	0.00020818
AAEL004021	AAEL004021	Glucose dehydrogenase [Source:UniProtKB/TrEMBL;Acc:A0A1S4F6C5]	-1.5090	0.05152906
AAEL002784	AAEL002784	zinc finger protein	-1.4973	0.00449399
AAEL004543	AAEL004543	trypsin, putative	-1.4837	0.02919615
AAEL017335	GPRGHP2	GPCR Growth Hormone Releasing Hormone Family	-1.4353	0.02973076
AAEL000242	AAEL000242	voltage-gated potassium channel	-1.4314	0.04602447
AAEL003657	AAEL003657	zinc finger protein	-1.4281	0.01598936
AAEL011354	AAEL011354	zinc finger protein	-1.3958	0.00528312
AAEL013374	AAEL013374	disconnected protein	-1.3950	0.00329504
AAEL015472	AAEL015472	catrin, putative	-1.3932	0.06292019
AAEL003897	AAEL003897	DNA repair protein xp-c / rad4	-1.3887	0.00324441
AAEL005744	AAEL005744	DEAD box ATP-dependent RNA helicase	-1.3845	0.02362442
AAEL011118	AAEL011118	Dual specificity protein kinase [Source:UniProtKB/TrEMBL;Acc:A0A1S4FSD2]	-1.3825	0.02094015
AAEL007876	AAEL007876	tyrosine protein kinase with ig domain	-1.3816	0.04140517
AAEL012499	AAEL012499	Histone H2A [Source:UniProtKB/TrEMBL;Acc:Q16LW9]	-1.3811	0.06399188
AAEL013406	AAEL013406	venom allergen	-1.3547	0.05408274
AAEL013380	AAEL013380	Adult cuticle protein, putative [Source:UniProtKB/TrEMBL;Acc:A0A1S4FZC4]	-1.3510	1.85E-05
AAEL005297	AAEL005297	guanine nucleotide exchange factor	-1.3387	0.044689
AAEL003470	ppk305	pickpocket 305	-1.3346	0.0891674
AAEL014550	AAEL014550	homeobox protein pknox1	-1.3346	0.07046153
AAEL010788	AAEL010788	metalloprotease	-1.3316	0.00076014
AAEL004371	AAEL004371	SWI/SNF related matrix associated actin dependent regulator of chromatin subfamily B member 1 [Source:UniProtKB/TrEMBL;Acc:A0A1S4F7F7]	-1.3295	0.00499854

Gene ID	Gene Name	Description	log2FC	p-adj*
AAEL013338	AAEL013338	lethal(2)essential for life protein, l2efl	-1.3223	0.02979345
AAEL008124	AAEL008124	possible RNA methyltransferase, putative	-1.3213	0.00084824
AAEL003624	AAEL003624	sodium/chloride dependent amino acid transporter	-1.3109	0.01888836
AAEL008021	AAEL008021	specifically Rac-associated protein, putative	-1.2889	0.01058611
AAEL004700	AAEL004700	cdkl1/4	-1.2747	0.01782479
AAEL008161	AAEL008161	abnormal oocyte, putative	-1.2574	0.00718728
AAEL009077	ALP1	Alkaline phosphatase [Source:UniProtKB/TrEMBL;Acc:A0A0P6JST2]	-1.2542	0.05899002
AAEL005064	CLIPB5	Clip-Domain Serine Protease family B.	-1.2423	0.02976933
AAEL003149	AAEL003149	F-box domain-containing protein [Source:UniProtKB/TrEMBL;Acc:A0A1S4F3V8]	-1.2327	0.00229875
AAEL007046	AAEL007046	mitochondrial brown fat uncoupling protein	-1.2320	0.05189952
AAEL001257	AAEL001257	sugar transporter	-1.2199	0.00311979
AAEL027536	AAEL027536	Putative alpha amylase catalytic domain family found in maltase [Source:UniProtKB/TrEMBL;Acc:A0A0P6IY06]	-1.2101	0.06798188
AAEL018312	AAEL018312	Structure-specific endonuclease subunit SLX1 homolog [Source:UniProtKB/TrEMBL;Acc:A0A1S4G7M6]	-1.2009	0.07460683
AAEL009924	AAEL009924	type II keratin, putative	-1.1978	0.04636698
AAEL008620	AAEL008620	D7 protein, putative	-1.1892	0.08229732
AAEL010881	AAEL010881	netrin receptor (unc5)	-1.1777	0.07928709
AAEL014051	AAEL014051	nop14	-1.1766	0.0691317
AAEL005383	AAEL005383	Adenosine monophosphate-protein transferase FICD homolog (EC 2.7.7.n1)	-1.1736	0.05629759
AAEL011965	AAEL011965	nuclear lamin L1 alpha, putative	-1.1654	0.04611012
AAEL011470	AAEL011470	cis,cis-muconate transport protein MucK, putative	-1.1646	0.07064081

Gene ID	Gene Name	Description	log2FC	p-adj*
AAEL011622	AAEL011622	serine protease, putative	-1.1634	0.04503562
AAEL011969	AAEL011969	protein-s isoprenylcysteine o-methyltransferase	-1.1550	0.01819871
AAEL006985	AAEL006985	dopachrome-conversion enzyme (DCE), putative	-1.1508	0.07868868
AAEL009846	AAEL009846	Enn protein, putative	-1.1420	0.03976897
AAEL011892	AAEL011892	receptor for activated C kinase, putative	-1.1362	0.04310771
AAEL002250	AAEL002250	terminal deoxycytidyl transferase rev1	-1.1356	0.00430004
AAEL010711	AAEL010711	eph receptor tyrosine kinase	-1.1332	0.0307846
AAEL011858	AAEL011858	transcription factor IIIA, putative	-1.1172	0.00334953
AAEL013554	CYP4J14	cytochrome P450	-1.1139	0.05964032
AAEL022478	AAEL022478	Putative transcriptional repressor protein yy1 [Source:UniProtKB/TrEMBL;Acc:A0A0P6IYK9]	-1.1033	0.09384216
AAEL010370	AAEL010370	aldehyde oxidase	-1.0995	0.07904213
AAEL010468	AAEL010468	DNA-directed RNA polymerase I, 12kD-subunit, putative	-1.0971	0.00128582
AAEL008576	AAEL008576	tRNA-splicing endonuclease subunit Sen34 [Source:UniProtKB/TrEMBL;Acc:A0A1S4FJZ8]	-1.0886	0.01400004
AAEL004853	AAEL004853	glycerol kinase	-1.0810	0.00107696
AAEL003676	AAEL003676	myosin I homologue, putative	-1.0798	0.04503562
AAEL008316	AAEL008316	mitotic spindle assembly checkpoint protein mad2	-1.0796	0.09095659
AAEL007853	AAEL007853	lupus la protein (Sjogren syndrome type B antigen) (La/SS-B), putative	-1.0689	0.06639301
AAEL014238	AAEL014238	aromatic amino acid decarboxylase	-1.0631	0.09614662
AAEL005312	AAEL005312	LRRcap domain-containing protein [Source:UniProtKB/TrEMBL;Acc:A0A1S4FA80]	-1.0592	0.09614662
AAEL009542	AAEL009542	Putative ribonucleasep/mrp protein subunit pop1 [Source:UniProtKB/TrEMBL;Acc:A0A0P6JS03]	-1.0554	0.04692902
AAEL010968	AAEL010968	Queuine tRNA-ribosyltransferase subunit QTRTD1 homolog (EC	-1.0410	0.03967919

Gene ID	Gene Name	Description	log2FC	p-adj*
		2.4.2.29)(Queuine tRNA-ribosyltransferase domain-containing protein 1)		
AAEL002978	AAEL002978	leucyl aminopeptidase, putative	-1.0400	0.07021345
AAEL006827	CYP12F8	cytochrome P450	-1.0354	0.0994708
AAEL001370	AAEL001370	muscle-specific tyrosine kinase receptor	-1.0339	0.07888516
AAEL024373	AAEL024373	Esr1 protein [Source:UniProtKB/TrEMBL;Acc:A0A1S4FP55]	-1.0302	0.0978428
AAEL010942	AAEL010942	kinesin-like protein KIF3A	-1.0283	0.02533003
AAEL005943	AAEL005943	ser/thr kinase stk11 (lkb1)	-1.0275	0.04809177
AAEL004955	AAEL004955	FAR1 domain-containing protein [Source:UniProtKB/TrEMBL;Acc:A0A1S4F947]	-1.0242	0.08744928
AAEL009090	AAEL009090	U6 snRNA phosphodiesterase [Source:UniProtKB/TrEMBL;Acc:A0A1S4FL98]	-1.0219	0.06119191
AAEL001169	AAEL001169	ribosome biogenesis protein BOP1 homolog	-1.0202	0.02282317
AAEL013014	AAEL013014	zinc finger protein	-1.0143	0.0656051
AAEL008878	AAEL008878	diacylglycerol o-acyltransferase	-1.0140	0.08411938
AAEL009285	AAEL009285	DEAD box ATP-dependent RNA helicase	-1.0116	0.04678077
AAEL014830	AAEL014830	cytochrome P450	-1.0095	0.00715786
AAEL001016	AAEL001016	zinc finger protein	-1.0079	0.00085038
AAEL000800	AAEL000800	microsomal dipeptidase	-1.0078	0.04292839
AAEL002948	AAEL002948	frataxin, putative	-1.0067	0.00666839

*adjusted by Benjamini-Hochberg correction method

Table S6. List of significantly upregulated annotated genes in Field-G2 samples relative to UPLB-UNXP-G122 samples from DESeq2 v.1.46.0 in R Studio v.2024.12.0+467

Gene ID	Gene Name	Description	log2FC	p-adj*
AAEL012664	AAEL012664	prolylcarboxypeptidase, putative	7.8486	0.00011338

Gene ID	Gene Name	Description	log2FC	p-adj*
AAEL004275	AAEL004275	Osiris, putative [Source:UniProtKB/TrEMBL;Acc:A0A1S4F763]	6.9338	0.00439859
AAEL002138	AAEL002138	triacylglycerol lipase, putative	6.2088	0.00435211
AAEL013161	AAEL013161	heat shock protein, putative	5.5402	0.00396986
AAEL007602	AAEL007602	trypsin, putative	5.3016	0.01028934
AAEL015363	AAEL015363	cuticle protein, putative	4.7502	0.01864312
AAEL007684	AAEL007684	tektin, putative	4.744	0.00023968
AAEL000029	AAEL000029	dopachrome-conversion enzyme (DCE), putative	4.6549	1.09E-11
AAEL015163	AAEL015163	cuticle protein, putative	4.5495	2.19E-09
AAEL001054	GSTD4	glutathione S-transferase (GSTD4)	4.0721	0.00220675
AAEL001822	AAEL001822	glucosyl/glucuronosyl transferases	3.9909	0.00208735
AAEL012700	AAEL012700	ATP-binding cassette sub-family A member 3, putative	3.8223	0.00021276
AAEL014700	AAEL014700	sugar transporter	3.5735	8.02E-07
AAEL014617	CYP9J28	cytochrome P450	3.4977	0.00036566
AAEL011169	AAEL011169	hexamerin 2 beta	3.4493	2.09E-06
AAEL013757	AAEL013757	hexamerin 2 beta	3.4337	3.78E-05
AAEL002246	AAEL002246	cuticle protein, putative	3.3454	0.01301852
AAEL000075	Gr9	gustatory receptor Gr9	3.22	0.00057868
AAEL000283	CTLMA16	C-Type Lectin (CTL) - mannose binding.	3.0923	0.00105994
AAEL001301	AAEL001301	CRAL-TRIO domain-containing protein [Source:UniProtKB/TrEMBL;Acc:A0A1S4EYD7]	3.019	3.38E-05
AAEL014613	CYP9J24	cytochrome P450	2.9355	0.01554319
AAEL013385	AAEL013385	brain chitinase and chia	2.8671	2.05E-08
AAEL014893	CYP6BB2	cytochrome P450	2.8292	7.41E-06
AAEL011822	AAEL011822	gamma glutamyl transpeptidases	2.8066	0.0046361

Gene ID	Gene Name	Description	log2FC	p-adj*
AAEL012072	AAEL012072	Histone H3 [Source:UniProtKB/TrEMBL;Acc:Q16N56]	2.7828	0.00943033
AAEL006429	AAEL006429	trypsin	2.7632	0.00011221
AAEL013018	OBP56	odorant binding protein OBP56	2.721	1.38E-08
AAEL009793	AAEL009793	cuticle protein, putative	2.6507	0.00552434
AAEL007669	AAEL007669	oxidoreductase	2.5744	0.00223435
AAEL011292	AAEL011292	protease m1 zinc metalloprotease	2.536	1.23E-06
AAEL003049	AAEL003049	pupal cuticle protein 78E, putative	2.526	0.00646796
AAEL014579	AAEL014579	trypsin, putative	2.4515	0.00102094
AAEL001312	CYP9M6	Cytochrome P450 monooxygenase CYP9M6v2 [Source:UniProtKB/TrEMBL;Acc:X5HYQ8]	2.4208	1.02E-10
AAEL000878	AAEL000878	cuticle protein, putative	2.4155	1.51E-05
AAEL006797	AAEL006797	F-box/leucine rich repeat protein	2.3918	0.00367553
AAEL009124	CYP6N12	cytochrome P450	2.3668	0.00064414
AAEL014609	CYP9J26	cytochrome P450	2.2487	0.00057319
AAEL006421	AAEL006421	trypsin, putative	2.2228	0.01913293
AAEL003809	AAEL003809	sugar transporter	2.1659	0.02337477
AAEL011798	AAEL011798	allergen, putative	2.1543	1.67E-07
AAEL013449	AAEL013449	metalloproteinase, putative	2.1385	0.00645587
AAEL006414	AAEL006414	trypsin	2.1343	0.0043809
AAEL006353	AAEL006353	sulfotransferase (sult)	2.1187	7.87E-05
AAEL002309	TPX4	Thioredoxin Peroxidase.	2.1159	0.00030553
AAEL005776	Orco	odorant receptor obligate co-receptor (Orco)	2.0859	2.32E-07
AAEL005687	AAEL005687	protein serine/threonine kinase, putative	2.0648	0.00166232
AAEL005374	SCRB1	Class B Scavenger Receptor (CD36 domain)	2.0489	3.25E-07
AAEL002688	AAEL002688	glucosyl/glucuronosyl transferases	2.0471	0.00036878

Gene ID	Gene Name	Description	log2FC	p-adj*
AAEL001318	AAEL001318	CRAL/TRIO domain-containing protein	2.0272	0.01805229
AAEL015631	AAEL015631	asparagine synthetase	2.0225	0.00015231
AAEL012383	AAEL012383	protein serine/threonine kinase, putative	2.0206	4.11E-05
AAEL014684	CYP6F3	cytochrome P450	1.9883	0.00121463
AAEL010393	AAEL010393	ferritin subunit, putative	1.977	0.02929916
AAEL006902	AAEL006902	serine-type enodpeptidase,	1.9587	0.00061584
AAEL006276	AAEL006276	maltose phosphorylase	1.9499	1.74E-05
AAEL014615	CYP9J23	cytochrome P450	1.9243	7.00E-07
AAEL009127	CYP6M11	cytochrome P450	1.9137	0.01048811
AAEL006327	AAEL006327	sulfotransferase (sult)	1.9111	0.00078871
AAEL000085	AAEL000085	pupal cuticle protein, putative	1.9039	0.05170392
AAEL025608	AAEL025608	Putative cuticle protein [Source:UniProtKB/TrEMBL;Acc:A0A0P6IXJ4]	1.8756	0.00746332
AAEL012903	AAEL012903	sugar transporter	1.8737	0.03271856
AAEL000057	TOLL5B	Toll-like receptor	1.8559	0.01094451
AAEL011263	AAEL011263	phosphatidylethanolamine-binding protein	1.8362	1.50E-07
AAEL010472	AAEL010472	helix-loop-helix protein hen	1.8302	8.99E-10
AAEL014137	CLIPB25	Clip-Domain Serine Protease family B.	1.8006	0.00114132
AAEL014412	CYP304B2	cytochrome P450	1.7951	3.98E-21
AAEL009748	AAEL009748	2-hydroxyphytanoyl-coa lyase [Source:UniProtKB/TrEMBL;Acc:Q16UY3]	1.7768	1.63E-19
AAEL002092	AAEL002092	cuticle protein, putative	1.7585	0.01773105
AAEL011499	OBP47	odorant binding protein OBP47	1.745	0.00566161
AAEL008767	AAEL008767	serine protease	1.7376	0.0036154
AAEL009760	AAEL009760	Niemann-Pick Type C-2, putative	1.7223	1.48E-06

Gene ID	Gene Name	Description	log2FC	p-adj*
AAEL005353	AAEL005353	Sugar transporter SWEET [Source:UniProtKB/TrEMBL;Acc:Q17A97]	1.7191	2.03E-09
AAEL000678	AAEL000678	alpha-amylase	1.7103	0.02314259
AAEL005412	AAEL005412	annexin x	1.7069	0.01317275
AAEL006403	AAEL006403	trypsin-beta, putative	1.6863	0.02023664
AAEL011633	AAEL011633	fibrinogen and fibronectin	1.6598	1.40E-05
AAEL012101	AAEL012101	tubulin alpha chain	1.6598	0.00361773
AAEL008045	AAEL008045	hexamerin 2 beta	1.6543	0.00872873
AAEL004724	AAEL004724	Carboxylic ester hydrolase (Fragment) [Source:UniProtKB/TrEMBL;Acc:Q17C44]	1.6346	7.52E-06
AAEL002693	AAEL002693	venom allergen	1.6229	7.16E-06
AAEL008456	AAEL008456	alpha-amylase	1.6054	0.03715475
AAEL001059	GSTD3	glutathione S-transferase (GSTD3)	1.6051	0.04016209
AAEL000651	AAEL000651	alpha-amylase	1.5823	0.00149752
AAEL006805	CYP9J2	cytochrome P450	1.5214	0.0071009
AAEL004369	AAEL004369	alpha-glucosidase	1.5095	0.02171281
AAEL006179	AAEL006179	tubulin alpha chain	1.5062	3.45E-07
AAEL000533	CTL16	C-Type Lectin (CTL16)	1.504	0.00178153
AAEL009449	OBP39	odorant binding protein (OBP39)	1.4991	0.01274568
AAEL005138	AAEL005138	glucosyl/glucuronosyl transferases	1.4963	0.00028438
AAEL000012	Gr6	gustatory receptor Gr6	1.4868	0.03813747
AAEL010286	AAEL010286	leucine-rich transmembrane protein	1.4853	0.00191395
AAEL010157	AAEL010157	microsomal glutathione s-transferase	1.4826	0.00593581
AAEL015567	OBP63	odorant binding protein OBP63	1.4644	0.00232869
AAEL003589	AAEL003589	transcription factor, putative	1.4434	3.22E-05
AAEL003012	AAEL003012	protease m1 zinc metalloprotease	1.4213	0.0299813
AAEL002520	AAEL002520	apolipoprotein D, putative	1.4162	0.03813747
AAEL014246	AAEL014246	glucosyl/glucuronosyl transferases	1.4143	0.01721539

Gene ID	Gene Name	Description	log2FC	p-adj*
AAEL008749	AAEL008749	udp-glucuronosyltransferase	1.4108	0.03253533
AAEL003954	AAEL003954	juvenile hormone-inducible protein, putative	1.4101	0.0002639
AAEL000025	AAEL000025	phosphoenolpyruvate carboxykinase	1.4045	0.03954797
AAEL014541	AAEL014541	maltose phosphorylase	1.3993	0.01571128
AAEL003971	AAEL003971	CHK domain-containing protein [Source:UniProtKB/TrEMBL;Acc:A0A1S4F696]	1.3948	0.0002297
AAEL009561	AAEL009561	apolipoprotein D, putative	1.3846	0.00150743
AAEL003504	AAEL003504	emx homeobox protein	1.365	0.00046961
AAEL005026	AAEL005026	ATP-dependent bile acid permease	1.3277	0.03505927
AAEL000667	AAEL000667	alpha-amylase	1.3268	0.00142359
AAEL008397	GPXH2	Glutathione peroxidase [Source:UniProtKB/TrEMBL;Acc:Q5K6H6]	1.3252	1.20E-05
AAEL008660	AAEL008660	Fig-alpha [Source:UniProtKB/TrEMBL;Acc:A0A1S4FK79]	1.3167	0.00484399
AAEL006077	AAEL006077	allatostatin receptor	1.312	0.03856217
AAEL001985	AAEL001985	protein serine/threonine kinase, putative	1.3089	0.01028934
AAEL006454	OBP1	odorant binding protein (OBP1)	1.3014	0.00091952
AAEL008817	AAEL008817	hexamerin 2 beta	1.2762	0.03187132
AAEL002141	AAEL002141	endothelin-converting enzyme	1.2758	0.00314092
AAEL009328	AAEL009328	hydroxyacyl dehydrogenase	1.2742	0.00552434
AAEL002031	CYP12F7	cytochrome P450	1.2723	6.46E-05
AAEL017136	CYP325V1	cytochrome P450	1.2707	0.01837131
AAEL014614	AAEL014614	cytochrome P450	1.2647	0.00223254
AAEL009384	AAEL009384	fibrinogen and fibronectin	1.2627	0.07542878
AAEL005109	AAEL005109	WD-repeat protein	1.255	0.00876261
AAEL000516	AAEL000516	juvenile hormone-inducible protein, putative	1.2538	0.00012962
AAEL006176	OBP27	odorant binding protein OBP27	1.2521	0.00054732

Gene ID	Gene Name	Description	log2FC	p-adj*
AAEL008090	AAEL008090	glutamate receptor, ionotropic, n-methyl d-aspartate epsilon (nmda epsilon)	1.2483	0.01956984
AAEL012377	OBP55	odorant binding protein OBP55	1.245	0.00681587
AAEL006784	CYP9J17	cytochrome P450	1.2203	0.04039322
AAEL006487	AAEL006487	sodium/solute symporter	1.2157	2.20E-05
AAEL012342	AAEL012342	lysosomal acid lipase, putative	1.2061	0.01769965
AAEL014618	AAEL014618	cytochrome P450	1.1998	0.01997796
AAEL008289	AAEL008289	pupal cuticle protein 78E, putative	1.1992	0.00095114
AAEL008342	AAEL008342	monocarboxylate transporter	1.1985	0.01148595
AAEL010381	AAEL010381	Glucosyl/glucuronosyl transferases [Source:UniProtKB/TrEMBL;Acc:A0A1S4FQK1]	1.1964	0.01634872
AAEL012776	AAEL012776	protease m1 zinc metalloprotease	1.1959	0.00152403
AAEL000556	CTL25	C-Type Lectin (CTL25)	1.1858	0.03684766
AAEL014605	CYP9J9	cytochrome P450	1.1572	0.00254444
AAEL005752	AAEL005752	lysosomal alpha-mannosidase (mannosidase alpha class 2b member 1)	1.1436	0.04542295
AAEL012762	CYP325N2	cytochrome P450	1.1422	0.00706923
AAEL007018	AAEL007018	udp-glucose 4-epimerase	1.1237	0.00125172
AAEL000087	AAEL000087	macroglobulin/complement	1.1234	0.00040232
AAEL006815	CYP9J16	cytochrome P450	1.1231	0.01073866
AAEL006919	AAEL006919	serine-type enodpeptidase,	1.1188	0.04159925
AAEL009881	AAEL009881	dynein heavy chain	1.112	0.00187889
AAEL012766	CYP325G2	cytochrome P450	1.1051	0.0530472
AAEL011730	AAEL011730	odorant-binding protein 99c, putative	1.0885	0.04077404
AAEL009128	CYP6M6	cytochrome P450	1.0735	0.03433628
AAEL007473	CYP6AH1	cytochrome P450	1.0721	0.03285364
AAEL014457	AAEL014457	neuronal cell adhesion molecule	1.0629	0.01914941

Gene ID	Gene Name	Description	log2FC	p-adj*
AAEL007224	LRIM22	leucine-rich immune protein (Coil-less)	1.0512	0.01484833
AAEL008202	AAEL008202	serine-type enodpeptidase,	1.0442	0.06754797
AAEL002877	AAEL002877	kinesin-like protein KIF17	1.044	0.02987998
AAEL001687	AAEL001687	adenylate cyclase	1.0377	0.05394573
AAEL000051	OBP3	odorant binding protein OBP3	1.0341	0.03117482
AAEL000762	LRIM19	leucine-rich immune protein (Coil-less)	1.0289	0.03880608
AAEL002005	CYP12F6	cytochrome P450	1.0286	0.00233423
AAEL006342	AAEL006342	epoxide hydrolase	1.0257	0.00129504
AAEL014297	AAEL014297	xenotropic and polytropic murine leukemia virus receptor xpr1	1.0255	0.00107756
AAEL004487	AAEL004487	valacyclovir hydrolase	1.0183	0.03754315
AAEL006425	AAEL006425	trypsin	1.0173	0.02264854
AAEL001938	AAEL001938	ATP-binding cassette sub-family A member 3, putative	1.016	0.00439859
AAEL007559	AAEL007559	guanyl-nucleotide exchange factor, putative	1.0062	0.00387014

*adjusted by Benjamini-Hochberg correction method

Table S7. List of significantly downregulated annotated genes in Field-G2 samples relative to UPLB-UNXP-G122 samples from DESeq2 v.1.46.0 in R Studio v.2024.12.0+467

Gene ID	Gene Name	Description	log2FC	p-adj*
AAEL012565	AAEL012565	lumbrokinase-3(1) precursor, putative	-6.2999	1.69E-05
AAEL013198	AAEL013198	ionotropic glutamate receptor-invertebrate	-3.8158	1.80E-07
AAEL003297	AAEL003297	alkaline phosphatase	-3.705	0.00037299
AAEL008474	AAEL008474	glycerol kinase	-3.4644	0.00120997
AAEL006975	AAEL006975	leucine aminopeptidase	-3.0042	0.01216335
AAEL013422	AAEL013422	odorant receptor 42a, putative	-2.8464	0.00436087
AAEL004028	AAEL004028	glucose dehydrogenase	-2.6766	0.00070026

Gene ID	Gene Name	Description	log2FC	p-adj*
AAEL008655	GPRVPR2	GPCR Vasopressin Family	-2.2243	9.79E-05
AAEL004021	AAEL004021	Glucose dehydrogenase [Source:UniProtKB/TrEMBL;Acc:A0A1S4F6C5]	-2.158	1.51E-05
AAEL002639	Rpl36-2	60S ribosomal protein L36 [Source:UniProtKB/TrEMBL;Acc:Q17HK5]	-2.1326	3.40E-10
AAEL010202	AAEL010202	trypsin	-2.005	0.0498268
AAEL007225	AAEL007225	dynein heavy chain	-1.8409	0.02779837
AAEL001674	AAEL001674	serine-type enodpeptidase,	-1.779	0.06346307
AAEL000242	AAEL000242	voltage-gated potassium channel	-1.6881	0.03277106
AAEL007040	AAEL007040	lozenge	-1.6327	0.00767614
AAEL007876	AAEL007876	tyrosine protein kinase with ig domain	-1.6311	0.00011692
AAEL007519	AAEL007519	oviductin	-1.5511	0.02993226
AAEL014066	AAEL014066	ubiquitin conjugating enzyme 7 interacting protein	-1.485	0.00253913
AAEL001257	AAEL001257	sugar transporter	-1.4831	0.00029269
AAEL026460	AAEL026460	Histone H3 [Source:UniProtKB/TrEMBL;Acc:Q16N56]	-1.4485	0.00132845
AAEL005373	GPROP12	pteropsin	-1.4282	0.04902243
AAEL015456	AAEL015456	ninjurin a	-1.4229	0.08079466
AAEL004883	AAEL004883	guanylate cyclase	-1.385	0.0005232
AAEL015472	AAEL015472	catrin, putative	-1.3372	0.05167449
AAEL006282	AAEL006282	tetraspanin, putative	-1.3266	0.00672269
AAEL010788	AAEL010788	metalloprotease	-1.3062	0.01200594
AAEL009178	GNBPB4	Gram-Negative Binding Protein (GNBP) or Beta-1 3-Glucan Binding Protein (BGBP).	-1.3025	0.05933564
AAEL018678	ND5	NADH dehydrogenase subunit 5	-1.2732	0.01100954
AAEL005049	AAEL005049	heterogeneous nuclear ribonucleoprotein	-1.2103	2.30E-09
AAEL007514	AAEL007514	Oviductin [Source:UniProtKB/TrEMBL;Acc:A0A1S4FGP8]	-1.1972	0.03730669

Gene ID	Gene Name	Description	log2FC	p-adj*
AAEL008427	AAEL008427	smad nuclear interacting protein	-1.1967	0.04831487
AAEL004259	AAEL004259	RNA-binding protein lin-28	-1.1263	0.06226825
AAEL003321	AAEL003321	F-box domain-containing protein [Source:UniProtKB/TrEMBL;Acc:A0A1S4F4A2]	-1.107	0.01977742
AAEL019789	AAEL019789	40S ribosomal protein S3a [Source:UniProtKB/Swiss-Prot;Acc:Q1HRR3]	-1.0933	0.01484833
AAEL005349	AAEL005349	Sugar transporter SWEET [Source:UniProtKB/TrEMBL;Acc:Q17A98]	-1.0605	0.03025625
AAEL014334	AAEL014334	lachesin, putative	-1.0345	0.05961081
AAEL011404	CTL19	C-Type Lectin (CTL19)	-1.0058	0.09334333

*adjusted by Benjamini-Hochberg correction method
