# Supplementary material for: Transcriptome Analysis of Deltamethrin Resistance Mechanisms in Aedes aegypti Populations from Metro Manila, Philippines
Source: Genes (Basel). 2026 Sep 10;17(9):1093. doi: 10.3390/genes17091093 (PMC13606281; doi:10.3390/genes17091093)
Supplement: Supplementary file 1 [file genes-17-01093-s001.zip › Figure_S4.pdf]

A

prolylcarboxypeptidase, putative (AAEL0121664)

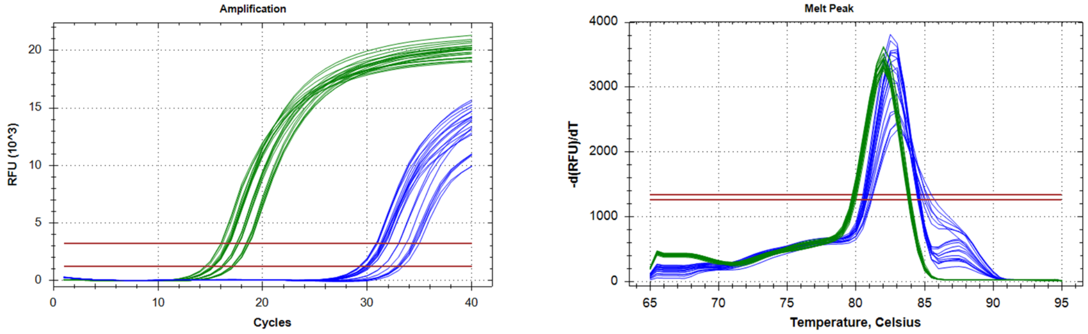

B

Osiris protein, putative (AAEL004275)

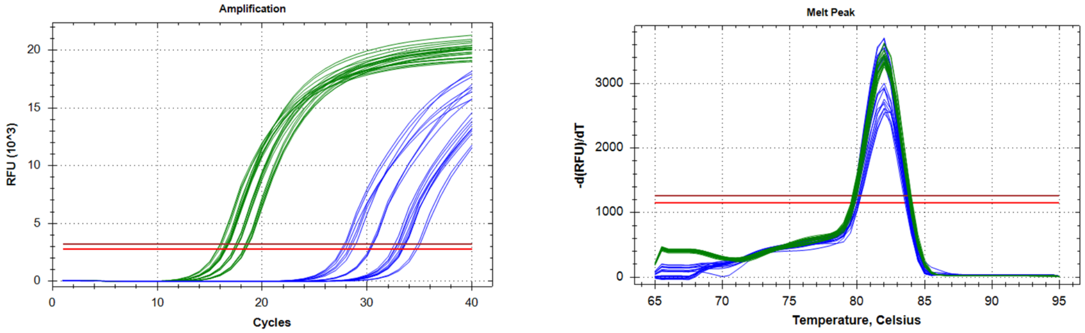

C

triacylglycerol lipase, putative (AAEL002138)

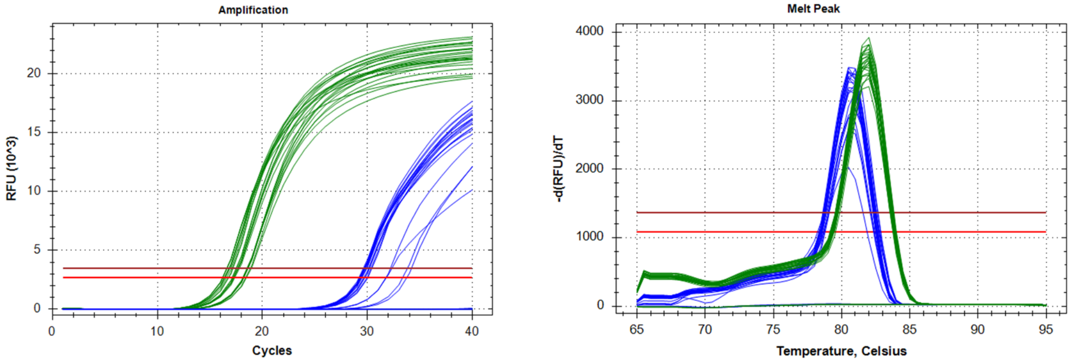

D

glutathione S-transferase delta 4 (GSTD4) (AAEL001054)

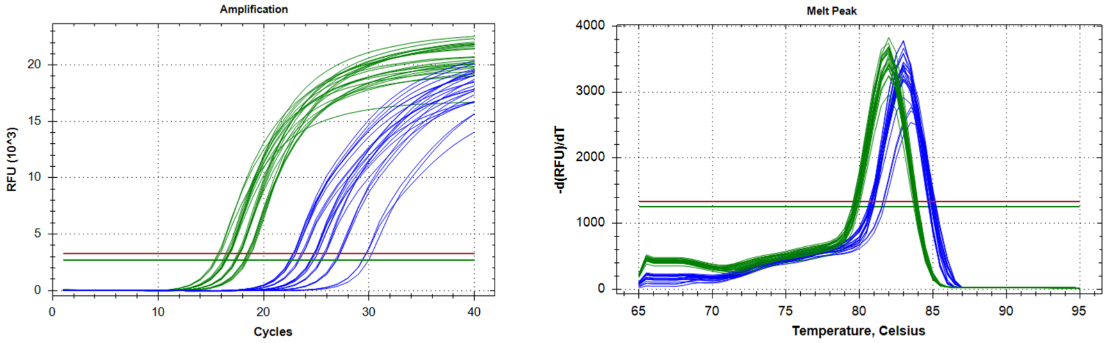

E

cytochrome P450 9J28 (CYP9J28) (AAEL014617)

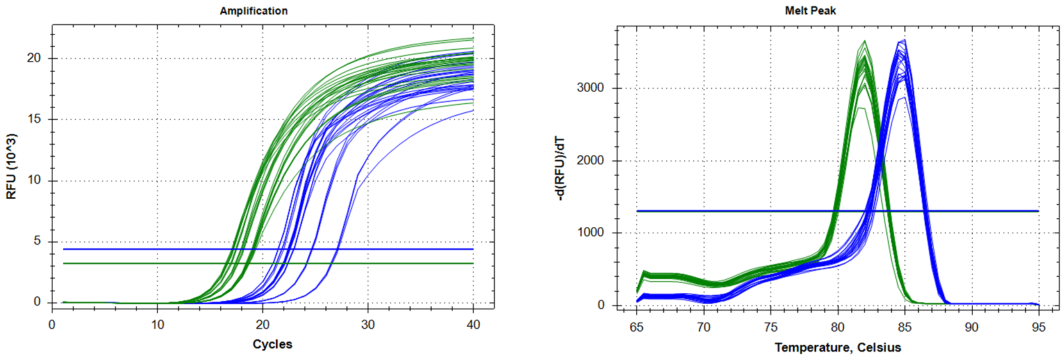

F

cytochrome P450 9J24 (CYP9J24) (AAEL014613)

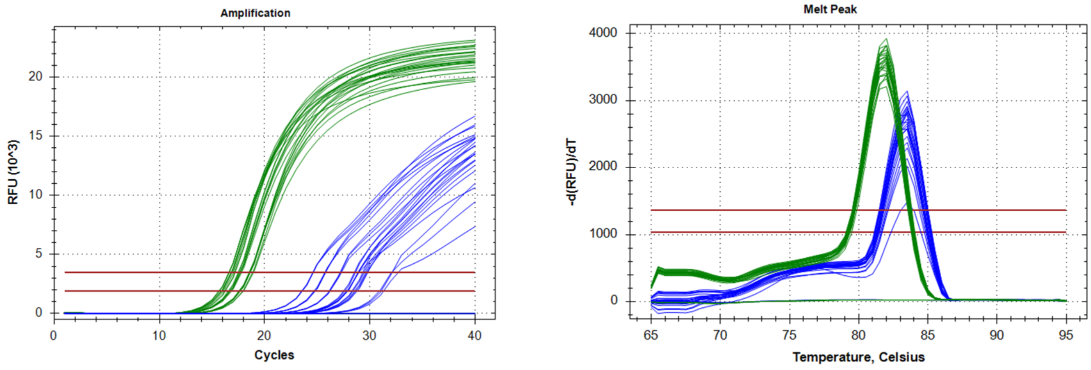

G

cytochrome P450 6BB2 (CYP6BB2) (AAEL014893)

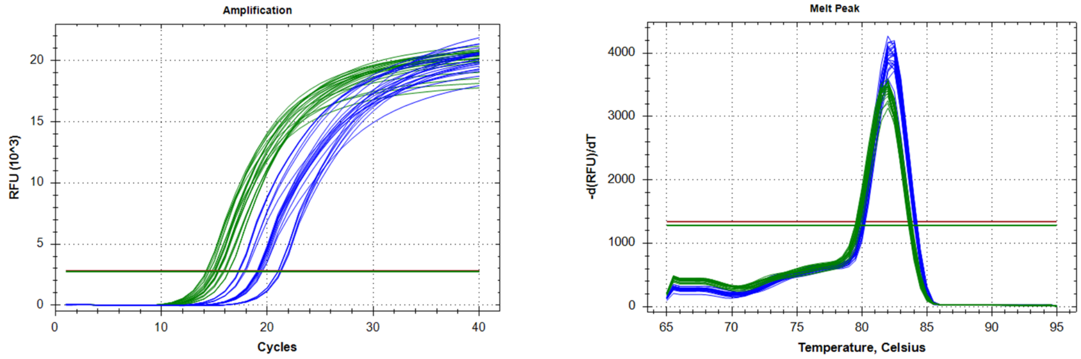

H

alkaline phosphatase (AAEL003297)

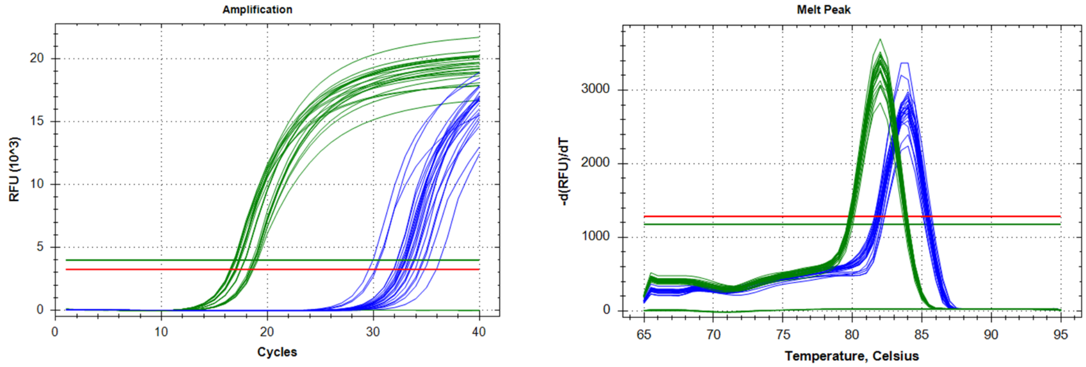

I

ionotropic glutamate receptor (AAEL013198)

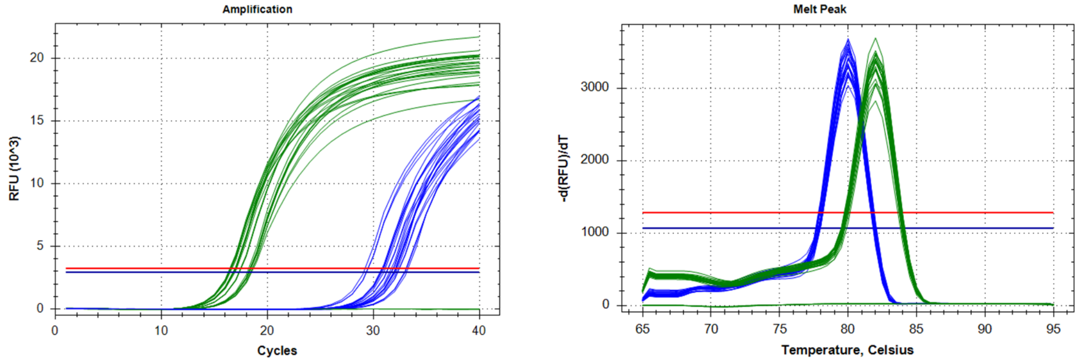

J

lumbrokinase-3(1) precursor, putative (AAEL012565)

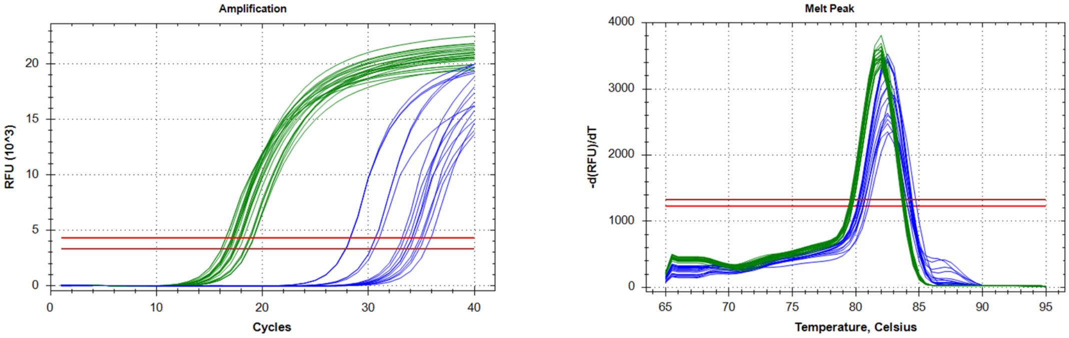

Figure S4. qPCR results from relative gene expression analysis of selected DEGs
