# Supplementary material for: Transcriptome Analysis of Deltamethrin Resistance Mechanisms in Aedes aegypti Populations from Metro Manila, Philippines
Source: Genes (Basel). 2026 Sep 10;17(9):1093. doi: 10.3390/genes17091093 (PMC13606281; doi:10.3390/genes17091093)
Supplement: Supplementary file 1 [file genes-17-01093-s001.zip › Figure_S3.pdf]

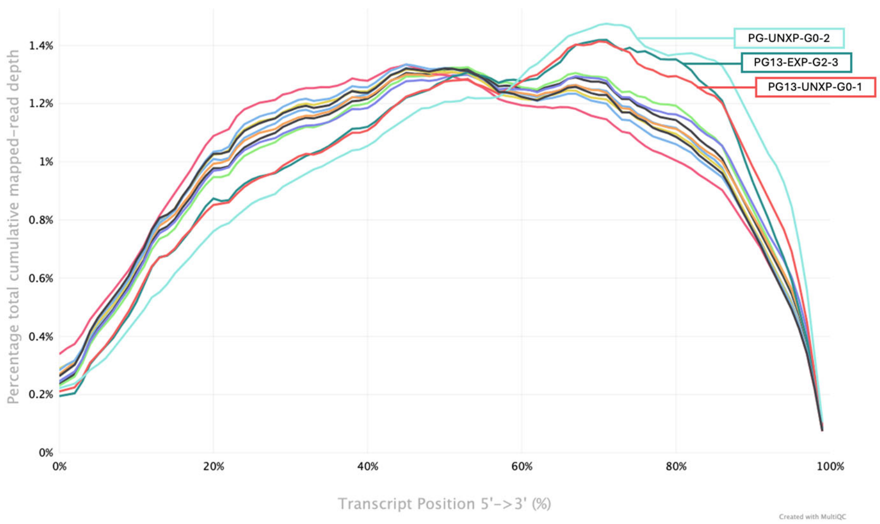

Figure S3. **Normalized read coverage profiles along genes (gene bodies)**. Read coverages in the 12 *Aedes aegypti* samples with observed 3' bias in the labeled curves from three (3) samples.
