# Supplementary material for: Transcriptome Analysis of Deltamethrin Resistance Mechanisms in Aedes aegypti Populations from Metro Manila, Philippines
Source: Genes (Basel). 2026 Sep 10;17(9):1093. doi: 10.3390/genes17091093 (PMC13606281; doi:10.3390/genes17091093)
Supplement: Supplementary file 1 [file genes-17-01093-s001.zip › Figure_S2.pdf]

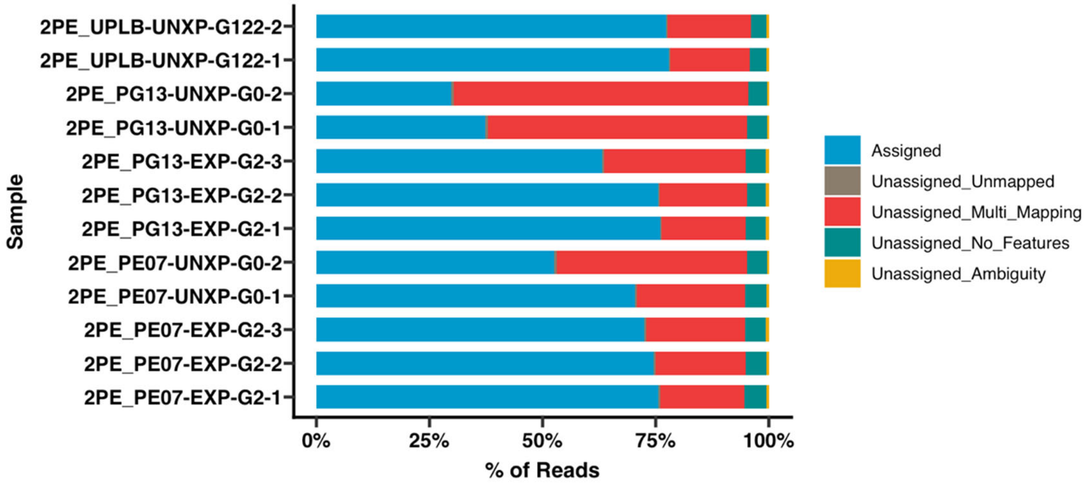

Figure S2. **Percentage of assigned and unassigned reads per sample**. Results from featureCounts v.2.0.8 showing the reads that are assigned and unassigned to the gene features from the annotation file of the AaegL5.3 assembly for *Aedes aegypti*.
